# Enhanced articular cartilage by human mesenchymal stem cells in enzymatically mediated transiently RGDS-functionalized collagen-mimetic hydrogels

**DOI:** 10.1016/j.actbio.2017.01.028

**Published:** 2017-03-15

**Authors:** Paresh A. Parmar, Jean-Philippe St-Pierre, Lesley W. Chow, Christopher D. Spicer, Violet Stoichevska, Yong Y. Peng, Jerome A. Werkmeister, John A.M. Ramshaw, Molly M. Stevens

**Affiliations:** a Department of Materials, Imperial College London, Exhibition Road, London SW7 2AZ, United Kingdom; b Department of Bioengineering, Imperial College London, Exhibition Road, London SW7 2AZ, United Kingdom; c Institute of Biomedical Engineering, Imperial College London, Exhibition Road, London SW7 2AZ, United Kingdom; d CSIRO Manufacturing, Bayview Avenue, Clayton, Victoria 3169, Australia; e Division of Biomaterials and Regenerative Medicine, Department of Medical Biochemistry and Biophysics, Karolinska Institutet, Scheeles väg 2, 17177 Stockholm, Sweden

**Keywords:** Hydrogel, Mesenchymal stem cell, Biodegradation, RGDS, Biomimetic material, Cartilage tissue engineering

## Abstract

Recapitulation of the articular cartilage microenvironment for regenerative medicine applications faces significant challenges due to the complex and dynamic biochemical and biomechanical nature of native tissue. Towards the goal of biomaterial designs that enable the temporal presentation of bioactive sequences, recombinant bacterial collagens such as Streptococcal collagen-like 2 (Scl2) proteins can be employed to incorporate multiple specific bioactive and biodegradable peptide motifs into a single construct. Here, we first modified the backbone of Scl2 with glycosaminoglycan-binding peptides and cross-linked the modified Scl2 into hydrogels via matrix metalloproteinase 7 (MMP7)-cleavable or non-cleavable scrambled peptides. The cross-linkers were further functionalized with a tethered RGDS peptide creating a system whereby the release from an MMP7-cleavable hydrogel could be compared to a system where release is not possible. The release of the RGDS peptide from the degradable hydrogels led to significantly enhanced expression of collagen type II (3.9-fold increase), aggrecan (7.6-fold increase), and SOX9 (5.2-fold increase) by human mesenchymal stem cells (hMSCs) undergoing chondrogenesis, as well as greater extracellular matrix accumulation compared to non-degradable hydrogels (collagen type II; 3.2-fold increase, aggrecan; 4-fold increase, SOX9; 2.8-fold increase). Hydrogels containing a low concentration of the RGDS peptide displayed significantly decreased collagen type I and X gene expression profiles, suggesting a major advantage over either hydrogels functionalized with a higher RGDS peptide concentration, or non-degradable hydrogels, in promoting an articular cartilage phenotype. These highly versatile Scl2 hydrogels can be further manipulated to improve specific elements of the chondrogenic response by hMSCs, through the introduction of additional bioactive and/or biodegradable motifs. As such, these hydrogels have the possibility to be used for other applications in tissue engineering.

**Statement of Significance:**

Recapitulating aspects of the native tissue biochemical microenvironment faces significant challenges in regenerative medicine and tissue engineering due to the complex and dynamic nature of the tissue. The ability to take advantage of, mimic, and modulate cell-mediated processes within novel naturally-derived hydrogels is of great interest in the field of biomaterials to generate constructs that more closely resemble the biochemical microenvironment and functions of native biological tissues such as articular cartilage. Towards this goal, the temporal presentation of bioactive sequences such as RGDS on the chondrogenic differentiation of human mesenchymal stem cells is considered important as it has been shown to influence the chondrogenic phenotype. Here, a novel and versatile platform to recreate a high degree of biological complexity is proposed, which could also be applicable to other tissue engineering and regenerative medicine applications.

## Graphical abstract

**Figure:**
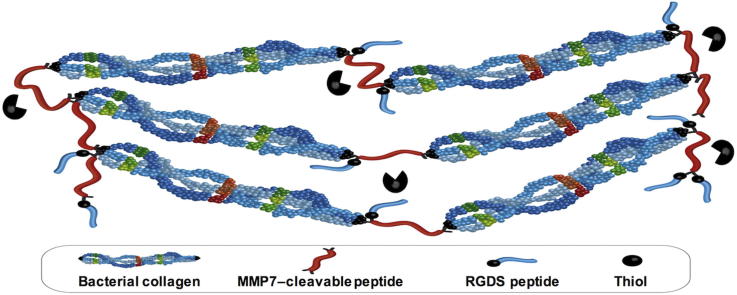

## 1 Introduction

Articular cartilage is a highly complex connective tissue that covers the surface of bones in synovial joints [1]. The unique spatial organization of the components of cartilage extracellular matrix is fundamental to its ability to carry out its biomechanical functions [2], [3]. Trauma to articular cartilage and/or disease of the joint can stimulate catabolic responses that disturb tissue homeostasis and can lead to progressive degeneration [4]. This is aggravated by the aneural and avascular nature of articular cartilage, combined with the limited ability of resident cells to migrate to sites of injury, which contribute to a restricted capacity for self-repair and regeneration [5]. Current clinical treatments for articular cartilage ailments, such as non-steroidal anti-inflammatory drugs [6], viscosupplementation [4], mosaicplasty [7], autologous chondrocyte implantation [8], microfracture [9], and periosteal transplantation [10], generally provide short-term pain relief and recovery of joint mobility to patients, but long-term benefits often remain elusive [3]. The repair tissue formed as a result of the surgical interventions listed here often does not exhibit the same biochemical composition as native tissue, leading to inferior biomechanical properties. Repair tissue is typically rapidly degenerated, ultimately leading to the failure of the intervention [4], thus requiring additional treatment and eventually total joint arthroplasty [11].

To overcome the limitations of current repair strategies, increasing efforts are aimed at the development of biomaterial scaffolds tailored to promote chondrogenesis, notably by providing instructive microenvironments that are reminiscent of aspects of the native pericellular matrix (PCM) [12], [13]. Cell-matrix interactions are dynamic; thus, biomaterials that present temporal changes to the presentation of bioactive cues, i.e., by harnessing the remodeling in response of resident cells, may allow improved control over complex processes such as chondrogenic differentiation [14].

Hydrogels are three-dimensional (3D) aqueous-based matrices that have been widely explored as scaffolds to encapsulate cells. Many attempts have been made to recapitulate aspects of the complex and dynamic cell-extracellular matrix (ECM) interactions present in articular cartilage and other tissues by incorporating specific bioactive and/or biodegradable components into hydrogel systems [15], [16], [17]. Biodegradable hydrogels in particular, have been extensively studied in cartilage repair applications to create space for newly deposited matrix [14]. Hydrogel degradation and ECM accumulation rates that are closely linked have been suggested to be fundamental to optimal tissue repair [13], [14].

Hydrogel biodegradability is often implemented through the incorporation of hydrolytically or enzymatically cleavable cross-linkers [14], [18]. Hydrolytic degradation of hydrogels can be partially tunable and has been shown to stimulate cell proliferation and ECM accumulation in cartilage tissue engineering [19]. However, the rate of degradation in such gels is more dependent on the macromer composition than on cell behavior and generally does not comply with cellular function. In contrast, enzymatically degradable hydrogels respond to changes in protease secretion by encapsulated cells, allowing for cell-mediated control over hydrogel degradation kinetics. Matrix metalloproteinases (MMPs) and other enzymes including plasmin [14] are commonly exploited for this purpose as they are involved in native tissue remodeling [20], [21], [22], [23], [24], [25]. The use of such enzymatic-degradation systems has also been shown to lead to improved cartilage ECM accumulation and elaboration [14], [17], [18], [21].

Scl2 proteins have recently been the subject of a number of studies as a potential alternative to mammalian collagens for tissue engineering applications [21], [26], [27], [28], [29], [30], [31], [32]. Scl2 proteins consist of a characteristic repeating (Gly–Xaa–Yaa)_n_ sequence arranged in a triple helical conformation, but lack the bioactive sites that mediate cell responses in mammalian collagens [33]. In contrast to mammalian collagens, Scl2 proteins are non-immunogenic, non-cytotoxic, and can be recombinantly produced in high yields with minimal batch to batch variation [32]. Additionally, the backbone of Scl2 helices can easily be altered to incorporate bioactive and/or biodegradable components via tethering or site-directed mutagenesis, in order to modulate cellular behavior [21], [34]. Previously, Scl2 proteins have been used to generate poly(ethylene glycol) (PEG)-Scl2 hybrid hydrogels functionalized with an integrin-binding sequence (GFPGER) to interact with smooth muscle and endothelial cells for vascular grafts [31]. Our group has recently developed Scl2-based scaffolds functionalized with glycosaminoglycan (GAG)-binding peptides and/or cross-linked by enzymatically-cleavable peptides, designed to drive the chondrogenic differentiation of hMSCs and degradation of the hydrogels [21], [34], [35].

Human mesenchymal stem cells (hMSCs) have been shown to benefit from the presence of fibronectin in the early stages of chondrogenic differentiation [18], [22], [23], [24]. Fibronectin gene expression levels are also up-regulated during these early stages of chondrogenic differentiation [24], [25]. The interaction of hMSCs with this extracellular protein via integrin-adhesive ligands is thought to affect cell-signaling and aid condensation and differentiation into chondrocytes [26]. More specifically, the arginine–glycine–aspartic acid (RGD) cell-adhesive sequence present on fibronectin has been shown to play an important role in initiating hMSC chondrogenesis [18]. However, in the later stages of chondrogenesis, fibronectin gene expression levels are down-regulated [26], [27], allowing complete differentiation of hMSCs towards chondrocytes. The RGD motif is often used to maintain hMSC viability in hydrogels that do not offer other inherent cell-adhesive motifs [18], [28], [29]. However, studies have demonstrated that the persistence of the RGD moiety can delay or even alter the chondrogenic differentiation of hMSCs, often leading to hypertrophy, as clearly demonstrated in a previous study [18]. Concentration and temporal presentation of this RGD moiety are thus important design criteria for the development of hydrogels that promote chondrogenic differentiation.

**Fig. 1:**
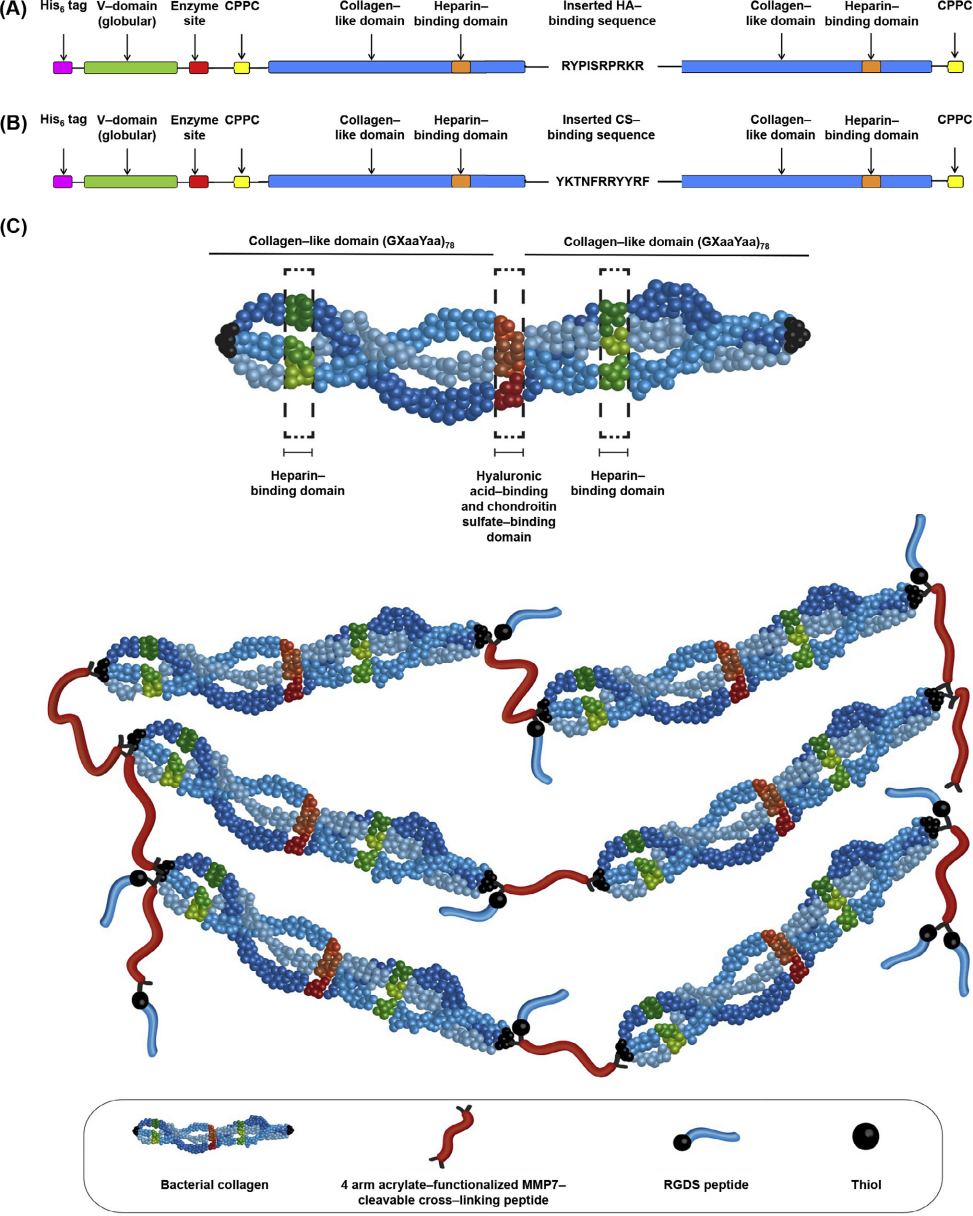
Schematic diagrams of the peptide-functionalized Scl2 proteins and resulting hydrogels. (A) Schematic diagram of the Scl2 protein containing heparin-binding and HA-binding peptide sequences. (B) Schematic diagram of the Scl2 protein containing heparin-binding and CS-binding peptide sequences. The ‘CPPC’ domains represent the amino acid sequences inserted at the N and C termini to aid stability of each construct. (C) Generalized structure of the peptide-functionalized Scl2 proteins and hydrogels. For clarity, the homotrimer chains are represented as different colors, and the triple helical motif has been opened out to show the three chains. The approximate locations of the heparin-binding sites are displayed in the construct. The 4-arm acrylate-functionalized MMP7-cleavable and non-cleavable ScrMMP7 peptides were conjugated to the Scl2 proteins via thiol-acrylate reactions to generate hydrogels. The cyclic RGDS peptide was conjugated to the 4-arm acrylate-functionalized MMP7-cleavable and non-cleavable ScrMMP7 peptides via thiol-acrylate reactions. It should be noted that there can be multiple different permutations for the cleaved RGDS peptide products i.e., with and without the Scl2 protein. Adapted from [35]. (For interpretation of the references to colour in this figure legend, the reader is referred to the web version of this article.)

In this work, we designed MMP7-cleavable hydrogels based on Scl2 functionalized within the backbone with GAG-binding peptides, using concepts from our previous work and presenting RGDS moieties that can also be released by the action of MMP7. We first modified the backbone of Scl2 to incorporate heparin (H), hyaluronic acid (HA), and chondroitin sulfate (CS)-binding sequences via site-directed mutagenesis (Fig. 1). Recent studies have shown the selected GAG-binding peptides to bind specifically and non-covalently to heparin, HA, and CS, respectively [36]. The inclusion of the HA-binding and CS-binding peptides was verified in our previous work [21] and was shown to enhance hMSC chondrogenesis. Heparin is present in articular cartilage and is known to encourage the recruitment of, and to form stable complexes with, growth factors such as TGF-β thus further aiding chondrogenesis in long-term culture [16], [19], [21]. To provide RGDS binding sites that have the potential to be enzymatically released from the hydrogel, we prepared MMP7-cleavable peptides with 25 or 50% (molar ratio) of the linker positions functionalized with RGDS and used these to cross-link Scl2 based hydrogels. We demonstrated the temporal release of RGDS from the hydrogels and investigated the effect of this release on chondrogenesis in hMSCs.

## 2 Materials and methods

### 2.1 Materials

Rink amide resin, Fmoc-protected amino acids, *N*,*N*-dimethylformamide (DMF), dichloromethane (DCM), 20% (v/v) piperidine in DMF, *O*-benzotriazole-*N*,*N*,*N*′,*N*′-tetramethyluronium-hexafluoro-phosphate (HBTU), and diisopropylethylamine (DIEA) were purchased from AGTC Bioproducts (UK). MMP7 fluorogenic substrate was purchased from Merck Millipore (UK). All primary and secondary antibodies used for immunohistochemistry were purchased from Abcam (UK). All other chemicals were purchased from Sigma–Aldrich (UK). All chemicals were used as provided by the manufacturers. Recombinant Scl2 proteins were expressed in *Escherichia coli* (*E*. *coli*) BL21-DE3 and purified as described in Section 2.3.

### 2.2 Peptide synthesis and purification

The MMP7-cleavable (PLELRA) and non-cleavable scrambled MMP7 (ScrMMP7; PALLRE) peptides (Fig. S1) were synthesized manually on a 2 mmol scale using standard Fmoc solid phase peptide synthesis techniques as previously described [36]. Two Fmoc-Lys(Mtt)-OH were also coupled to the peptides at the N- and C-termini, selectively deprotected with 5% (v/v) trifluoroacetic acid (TFA) in DCM, and reacted with an excess of acryloyl chloride to enable acrylate group functionalization. A cyclic RGDS peptide (GRGDSC) was synthesized on a 1 mmol scale on 2-chlorotrityl chloride resin (100–200 mesh; VWR) (Fig. S1).

### 2.3 *Streptoccocal collagen*-like 2 protein synthesis and purification

The gene constructs used were based on the DNA sequence for the fragment of the *Scl2.28* allele (Q8RLX7) of *Streptococcus pyogenes* encoding the combined globular and collagen-like portions of the *Scl2.28* protein, but lacking the C terminal attachment domain as previously described [32], [33], [37]. Constructs included an additional pepsin cleavage and spacer sequence, LVPRGSP, between the N terminal globular domain (V) and the following (Gly–Xaa–Yaa)_n_ collagen-like (CL) domain sequences. The construct prepared for this study (termed HHACS-Scl2) contained a HA-binding (RYPISRPRKR) or a CS-binding (YKTNFRRYYRF) sequence between two CL domains (Fig. 1). In addition, each of the two CL domains included heparin-binding (GRPGKRGKQGQK) sequences integrated within the triple helical structure, as previously described [32], [33], [37]. To stabilize the triple helix and allow for functionalization via thiol-acrylate chemistry, the N- and C-termini of this initial construct included an additional GGPCPPC sequence. This DNA sequence was synthesized commercially with codon optimization for expression in *E. coli* (GeneArt® Gene Synthesis, Germany). The sequences of the initial and final constructs were confirmed by sequencing prior to transformation and protein expression.

The final DNA sequences were sub-cloned into the pColdI (Takara Bio, Japan) vector systems for expression in *E. coli* (Fig. S2) in order to add an N-terminal His_6_-tag [32], [33], [37]. For protein production, a selected positive clone was transformed and then expanded in flask culture. The pColdI constructs were expressed in the *E. coli* BL21-DE3 strain. The selected positive clone was later confirmed using gene sequencing. Cells were grown in 2 × yeast extract–tryptone (YT) media with ampicillin (100 μg/mL) at 37 °C, with shaking at 200 rpm until the A600 absorbance reading reached an optical density in the range 3–6 A.U. Cells were then cooled to 25 °C and 1 mM isopropyl β-d-thiogalactopyranoside (IPTG) was added to induce protein expression. After 10 h incubation, cells were further cooled to 15 °C for 14 h, after which the cells were harvested by centrifugation (12,000*g*, 60 min) at 4 °C. For protein extraction, each 1 g of cell paste was resuspended in 20 mL of 20 mM sodium phosphate buffer at pH 8.0 and the cells ruptured by sonication on ice using a Misonix S4000 instrument with an Enhance Booster #1 probe [32], [33], [37]. Clarified lysate was obtained by centrifugation (12,000*g* for 30 min, 4 °C), adjusted to pH 2.2 and held at 4 °C for 16 h. Any precipitate that had formed was removed by centrifugation (12,000*g* for 30 min, 4 °C) and the supernatant, containing the collagen, was treated by pepsin (0.01 mg/mL) for 16 h at 4 °C. Collagen was concentrated and buffer exchanged into 20 mM sodium phosphate buffer, pH 8.0 using a 10 kDa cross-flow filtration membrane (Pall Life Sciences). Purity was verified by 12% (w/v) sodium dodecyl sulfate-polyacrylamide gel electrophoresis (SDS–PAGE) and matrix-assisted laser desorption spectroscopy (MALDI; Waters) [32], [33], [37].

### 2.4 Characterization of functionalized Scl2 proteins

Circular dichroism (CD) spectra of the HHACS-Scl2 protein in H_2_O were recorded on a Jasco J-715 spectropolarimeter controlled by the Jasco Spectra Manager software equipped with a Jasco PTC-348WI Peltier temperature control system using a quartz cuvette with a path length of 0.1 mm. The ellipticity at 220 nm was monitored [21], [31] as the sample temperature was increased from 25 to 40 °C with an average temperature slope of 10 °C/h to determine the thermal transitions. The ellipticity was normalized to the path length and number of amino acid residues and plotted against temperature.

The HHACS-Scl2 protein was analyzed using Fourier transform infrared (FTIR) spectroscopy on a Perkin Elmer Spectrum One spectrometer as previously described [21], [31]. FTIR spectra were taken with a scanning wavenumber range from 4000 to 650 cm^−1^.

### 2.5 Preparation of Scl2 hydrogels

To prepare hydrogels, the HHACS-Scl2 protein was re-suspended at 100 mg/mL in chondrogenic medium (high-glucose (4.5 g/L) Dulbecco’s Modified Eagle Medium (DMEM; Invitrogen, UK) supplemented with 0.1 mM dexamethasone, 1% (v/v) penicillin streptomycin, 50 μg/mL l-proline, 50 μg/mL ascorbate-2-phosphate, 1 × insulin-transferrin-selenium (ITS) Premix (BD Biosciences, UK), and 10 ng/mL TGF-β3 (Lonza, UK)) at room temperature. The acrylate-functionalized MMP7-cleavable and non-cleavable ScrMMP7 peptides were dissolved separately in 4 mM triethanolamine (TEA) in chondrogenic medium. In selected MMP7-cleavable and non-cleavable ScrMMP7 peptide samples, the cyclic RGDS peptide was dissolved and reacted (2 h, 37 °C, pH 7.4) to modify 25% or 50% (on a molar basis) of the acrylates and the resulting solutions were dialyzed against H_2_O overnight to remove unreacted by-products. For hydrogel formation, equimolar concentrations of the MMP7-cleavable and non-cleavable ScrMMP7 peptides were reacted (∼10 min, 37 °C, pH 7.4) to modify > 95% of the thiols on the HHACS-Scl2 protein. Prior to gelation, the resulting solutions were sterile-filtered and pipetted in 50 μL aliquots to generate 6 homogeneous hydrogel types: MMP7-HHACS-Scl2, MMP7-HHACS-lowRGDS-Scl2, MMP7-HHACS-highRGDS-Scl2, ScrMMP7-HHACS-Scl2, ScrMMP7-HHACS-lowRGDS-Scl2, and ScrMMP7-HHACS-highRGDS-Scl2. Following gelation, the wells were slowly topped up with chondrogenic medium.

### 2.6 Hydrogel characterization

#### 2.6.1 Morphological characterization

Hydrogels were imaged by multi-photon second harmonic generation (MP-SHG) in PBS using a Leica SP5 inverted microscope equipped with a MaiTai HP DeepSee multi-photon laser (Spectraphysics) on a 25× NA objective. Second harmonic signal was generated at 900 nm and detected on a photomultiplier tube (PMT) (435–465 nm).

#### 2.6.2 Mechanical characterization

Mechanical properties of the hydrogels were evaluated by oscillatory parallel plate rheology (Advanced Rheometer AR2000ex with AR Instrument Software fitted with a Peltier temperature control system, TA instruments). Samples were tested at 37 °C using an 8 mm diameter parallel steel plate. All samples were individually prepared immediately prior to testing. Two sequential sweeps were applied; (1) time sweep for 2 h at 0.1% strain and 6.28 rad/s angular frequency and (2) strain sweep from 0.01 to 100% at 6.28 rad/s angular frequency. For all samples, a compression load of 0.5 N was exerted during testing.

Hydrogels were also tested using dynamic mechanical analysis (DMA) in unconfined compression mode using a Bose Electroforce testing machine equipped with a 22.5 N load cell. For DMA, samples were incubated in PBS for 24 h prior to testing to ensure samples were in an equilibrium state and dimensions were measured in wet state using digital calipers. Samples were pre-loaded to 0.05 N, compressed to 10% strain at a crosshead speed of 0.5% strain/min, followed by a frequency sweep from 0.1 Hz to 10 Hz. The compressive moduli were calculated from the linear portion of the stress–strain curve.

#### 2.6.3 GAG binding

Binding and retention of specific GAGs to the heparin-binding, HA-binding, and CS-binding peptide sequences, incorporated into the Scl2 backbone, was evaluated using fluorescein isothiocyanate (FITC)-labeled heparin, HA, and CS (Creative PEGWorks, UK). Scl2 proteins were coated onto 96-well plates, incubated at 37 °C for 24 h, washed three times in PBS, incubated in 1% (w/v) bovine serum albumin (BSA) in PBS for 5 h and washed three times in PBS to prepare the assay plates. These were incubated in 0.5 mg/mL FITC-labeled heparin, HA, or CS for 24 h, washed three times in PBS to remove unbound fluorescent GAGs, and kept in PBS at 37 °C between measurements in the plate. The relative binding of fluorescently-labeled heparin, HA, or CS was normalized to the highest level of fluorescence intensity measured from each experiment to give a single 100% signal level that was used as a basis for normalization.

To study the release of heparin, HA, or CS from the hydrogels, the constructs were processed as described above for Scl2 proteins coated onto 96-well plates. After 1, 3, and 7 days, PBS was removed and fluorescence intensities of the supernatants were measured to evaluate GAG binding and retention. Samples were excited at 485 nm, and the fluorescence emission intensity was measured at 525 nm. The relative binding of heparin, HA, or CS was normalized to the highest level of fluorescence intensity at the first time point.

### 2.7 hMSC culture

Bone marrow-derived hMSCs were purchased from PromoCell GmbH (Germany). The multilineage differentiation capacity of the hMSCs was verified by PromoCell GmbH prior to purchase. hMSCs were seeded at 4000 cells per cm^2^ and cultured in mesenchymal stem cell growth medium (MSCGM) (PromoCell GmbH, Germany). hMSCs were incubated at 37 °C in a 5% CO_2_ atmosphere and the medium was changed every three days. The cells were harvested at approximately 80% confluency with 0.025% (w/v) trypsin-EDTA in PBS, centrifuged, and sub-cultured in MSCGM. Passage 6 hMSCs were used for all cell experiments.

### 2.8 Cell seeding and culture in hydrogels

hMSCs were homogeneously dispersed at 8 × 106 cells per mL in pre-made 100 mg/mL HHACS-Scl2 solutions containing chondrogenic medium as defined in Section 2.5. Hydrogels were formed in this solution as described above through the addition of cross-linking peptide. Aliquots (50 μL) of the mixture were pipetted in a non-tissue culture treated 48-well plate and allowed to gel for 30 min at 37 °C in a 5% CO_2_ atmosphere before slowly adding 1 mL of chondrogenic medium. Hydrogels were incubated at 37 °C in a 5% CO_2_ atmosphere for up to six weeks with the medium changed every three days.

### 2.9 Weight change, enzymatic activity assay, and RGDS peptide release

Hydrogel dry weights were measured over time in the absence of cells to evaluate their degradation *in vitro*. Hydrogels were incubated in chondrogenic medium for 24 h at 37 °C in a 5% CO_2_ atmosphere. The hydrogels were then incubated in chondrogenic medium with exogenous enzymes (30 ng/mL) at 37 °C in a 5% CO_2_ atmosphere for one week with medium changes and dry weight measurements after lyophilization taken daily. Percentage weight change was normalized to day 0. Degradation by recombinant human MMP1, MMP2, MMP7, and MMP13 (AnaSpec, USA) was tested against a negative control (chondrogenic medium alone) and a positive control (0.2 μg/mL trypsin). Next, cell-seeded hydrogels were incubated in chondrogenic medium at 37 °C in a 5% CO_2_ atmosphere for six weeks, and dry weight measurements after lyophilization were taken after 0, 1, 3, 7, 14, 21, 28, and 42 days of culture. Percentage weight change corresponding to the cumulative effect of cell proliferation, cartilage-like matrix deposition, and hydrogel degradation was normalized to day 0.

At each time point, 1 mL of medium was also removed, sterile-filtered, and analyzed for MMP7 activity using a fluorogenic MMP7 substrate assay according to the manufacturers’ instructions and compared to a negative control (chondrogenic medium) and a positive control (30 ng/mL recombinant human MMP7).

Release of the RGDS peptide from the hydrogels was evaluated over time in the absence of cells using cystamine-FITC as a model substrate in place of the RGDS peptide, coupled to the acrylates of the MMP7-cleavable and non-cleavable ScrMMP7 peptides. Cystamine-FITC was synthesized by reacting (2 h, room temperature, pH 7.4) cystamine dihydrochloride in an equimolar ratio with FITC (2 h in DCM) and purifying using reverse phase preparative HPLC. The hydrogels were incubated in phenyl red-free chondrogenic medium with exogenous recombinant human MMP7 (0.2 μg/mL) at 37 °C in a 5% CO_2_ atmosphere. After 0, 2, 4, 6, 8, 16, and 24 h, aliquots of the supernatants were removed and replaced with fresh medium, and their fluorescence intensities were measured to evaluate cystamine-FITC release. Samples were excited at 485 nm, and the fluorescence emission intensities were measured at 525 nm, respectively. Next, cell-seeded hydrogels were incubated in chondrogenic medium at 37 °C in a 5% CO_2_ atmosphere for seven days, and fluorescence intensity measurements of the supernatants were taken after 0, 1, 3, and 7 days of culture to evaluate the accumulative cystamine-FITC release, as an indicator of the RGDS peptide release from the hydrogels. Samples were excited at 485 nm, and the fluorescence emission intensities were measured at 525 nm, respectively.

### 2.10 Cell viability and metabolic activity

hMSC-seeded hydrogels were cultured for 0, 1, 3, 7, 14, 21, 28, and 42 days. After the culture period, the hydrogels were washed three times in PBS and analyzed for cell viability and metabolic activity. Cell viability was qualitatively assessed with a LIVE/DEAD® Viability/Cytotoxicity Kit (Molecular Probes, USA) according to the manufacturers’ instructions. Fluorescence confocal microscopy (Leica SP5 inverted microscope, Leica Microsystems, UK) was used to visualize live (calcein; green) and dead (ethidium homodimer-1; red) cells. The metabolic activity of cells in the hydrogels was quantified by the AlamarBlue® assay (Serotec, USA). This assay is based on the fluorescent signal output produced by metabolically active cells. Measurements were made at 570 nm and 600 nm. Cell-free hydrogels and empty wells were used as controls. All data were normalized to DNA content (described in Section 2.11.) at each time point.

### 2.11 DNA, sGAG, and hydroxyproline quantification

hMSC-seeded hydrogels were cultured for 0, 1, 3, 7, 14, 21, 28, and 42 days. After the culture period, the constructs were washed three times in PBS and digested individually in papain digest solution (2.5 units papain/mL, 5 mM cysteine HCl, 5 mM EDTA, pH 7.4, in PBS) at 60 °C for 24 h. Papain digests were stored at −20 °C until further analysis. Digests were assayed for DNA content using the Quant-iT™ PicoGreen® Kit (Invitrogen, USA) according to the manufacturers’ instructions. Measurements were made at 535 nm. The standard curve was generated with dsDNA (Invitrogen, USA). Sulfated GAG (sGAG) content was quantified using the Blyscan Kit (Biocolor, UK) according to the manufacturers’ instructions. Measurements were made at 656 nm. The standard curve was generated with bovine trachea chondroitin sulfate A. Total mammalian cell-derived collagen content was estimated by measuring the hydroxyproline content. Unlike mammalian collagens, bacterial collagens lack hydroxyproline, which enabled us to distinguish between the bacterial collagen in the hydrogel structure and new collagen deposition by the hMSCs. Papain-digested samples were hydrolyzed in 6 N HCl at 110 °C for 18 h. The hydroxyproline content of the hydrolysate was determined using the chloramine T-Ehrlich’s reagent assay and the color change quantified spectrophotometrically at 560 nm [38]. The standard curve was generated with l-hydroxyproline and a factor of 10 was used to convert from hydroxyproline to total collagen content estimation.

### 2.12 Histology and immunohistochemistry

After 0 and 42 days of culture, hMSC-seeded hydrogels were washed three times in PBS, fixed with 4% (v/v) paraformaldehyde (Electron Microscopy Sciences, USA) for 30 min at 4 °C, washed three times in PBS, permeabilized with 0.4% (v/v) Triton X-100 for 30 min, and washed again. Hydrogels were flash frozen in optimal cutting temperature medium (Tissue-Tek, Fisher Scientific) and cryosectioned at a thickness of 10 μm. Sections were transferred to treated slides (Superfrost Plus, Thermo Scientific) and allowed to adhere for 24 h at 4 °C. Slides were stained for deposited sGAG with Alcian Blue (AB; pH 2.5) and for cell nuclei and matrix with Haematoxylin and Eosin (H&E).

Immunohistochemical staining (IHC) was performed for collagen type I, collagen type II, collagen type X, SOX9, Runx2, and PPAR-γ. Samples were pre-treated with hydrogen peroxide, an avidin and biotin blocking kit (Vector Labs, UK), and blocked with 5% (v/v) goat serum. Primary antibodies were incubated overnight at 1:200 in 5% (v/v) goat serum, followed by goat anti-rabbit secondary antibody labeled with HRP at 1:100 for 1 h, stained with a 3,3′-diaminobenzidine (DAB) kit (Vector Labs, UK) for 10 min, and counter-stained with Haematoxylin. Rabbit IgG secondary antibody only and PBS negative controls were also tested. All stained sections were dehydrated, mounted with Histomount (Fisher Scientific, UK), and viewed on an Olympus BX51 microscope equipped with an Olympus DP70 camera.

### 2.13 Gene expression analysis

hMSC-seeded hydrogels were cultured for 0, 1, 3, 7, 14, 21, 28, and 42 days. After the culture period, the constructs were washed three times in PBS. Total RNA was isolated using a tissue ruptor (Qiagen, USA) to homogenize samples with RLT buffer after which QIAshredder columns (Qiagen, USA) and the RNeasy Mini Kit (Qiagen, USA) were used to extract the RNA according to the manufacturers’ instructions. QuantiTect® Reverse Transcription Kit (Qiagen, USA) and QuantiTect® SYBR Green polymerase chain reaction (PCR) Kit (Qiagen, USA) were used to perform reverse transcription and quantitative PCR (qPCR), respectively. Thermocycling and SYBR Green detection were performed on a Corbett Rotorgene 6000 (Qiagen, USA) with extension at 72 °C and denaturing at 95 °C. Annealing temperatures were primer specific. Data were analyzed using the ΔΔCt method [39]. The following primers were used: MMP7 (Forward 5′-GAGTGAGCTACAGTGGGAACA-3′ and Reverse 5′-CTATGACGCGGGAGTTTAACAT-3′), TIMP2 (Forward 5′-TGGACGTTGGAGGAAAGAAG-3′ and Reverse 5′-GGGCACAATGAAGTCACAGA-3′) at an annealing temperature of 52 °C, GAPDH (Quiagen, USA) (Forward 5′-TGGTATCGTGGAAGGACTCATGA-3′ and Reverse 5′-ATGCCAGTGAGCTTCCCGTTCAG-3′), COL1A1 (Forward 5′-CATTAGGGGTCACAATGGTC-3′ and Reverse 5′-TGGAGTTCCATTTTCACCAG-3′), COL2A1 (Forward 5′-CATCCCACCCTCTCACAGTT-3′ and Reverse 5′-GTCTCTGCCTTGACCCAAAG-3′), COL10A1 (Forward 5′-AATGCCTGTGTCTGCTTTTAC-3′ and Reverse 5′-ACAAGTAAAGATTCCAGTCCT-3′), and ACAN (Forward 5′-CACTGTTACCGCCACTTCCC-3′ and Reverse 5′-GACATCGTTCCACTCGCCCT-3′) at an annealing temperature of 60 °C, Runx2 (Forward 5′-CCGCCTCAGTGATTTAGGGC-3′ and Reverse 5′-GGGTCTGTAATCTGACTCTGTCC-3′) at an annealing temperature of 61 °C, and SOX9 (Forward 5′-AACGCCGAGCTCAGCAAG-3′ and Reverse 5′-ACGAACGGCCGCTTCTC-3′) at an annealing temperature of 62 °C.

### 2.14 Statistical analysis

All cell-related experiments were repeated three times with hMSCs from different donors and with an intra-experiment sample size of 3. Data are presented as means ± standard deviation (SD). Statistical significance was determined by performing analysis of variance (ANOVA) with Bonferroni correction and with a significance accepted at *p*-value < 0.05.

## 3 Results and discussion

### 3.1 Characterization of Scl2 proteins and acellular hydrogels

#### 3.1.1 Scl2 protein characterization

Changes in the secondary structure of the modified Scl2 protein as a consequence of the backbone functionalization were evaluated using CD (Fig. S3). A characteristic peak present at 220 nm in the CD spectra confirmed that the modified Scl2 adopted a triple helical structure as previously reported [21], [31]. In addition, no change in the thermal stability of the functionalized Scl2 triple helix was observed in the CD spectra at 37 °C. In accordance with our previous findings, the addition of cysteine residues at the N- and C-termini of the Scl2 construct likely resulted in the stabilization of the triple helical backbone through interchain disulfide formation [34], [35], [40]. Subsequent cross-linking of the functionalized Scl2 protein into hydrogels via the MMP7-cleavable and non-cleavable ScrMMP7 peptides would likely stabilize the backbone further [32]. It is likely that the cross-linking peptides interlocked individual Scl2 chains together thereby minimizing their mobility and denaturation.

FTIR spectroscopy confirmed addition of the MMP7-cleavable and scrambled non-cleavable ScrMMP7 cross-linking peptides to the Scl2 helix, through thiol-ene conjugation (Fig. S4). The FTIR spectra exhibited IR transmittance peaks at 1630 cm^−1^ (C=O) that were assigned to the amide functional group present on the Scl2 protein in all samples and used for normalization. The peak at 1110 cm^−1^ (C–S–C) assigned to the thioether functional group, present after peptide cross-linking, was observed in all samples except the non-cross-linked HHACS-Scl2 control.

#### 3.1.2 Gag-binding characteristics of functionalized Scl2

**Fig. 2:**
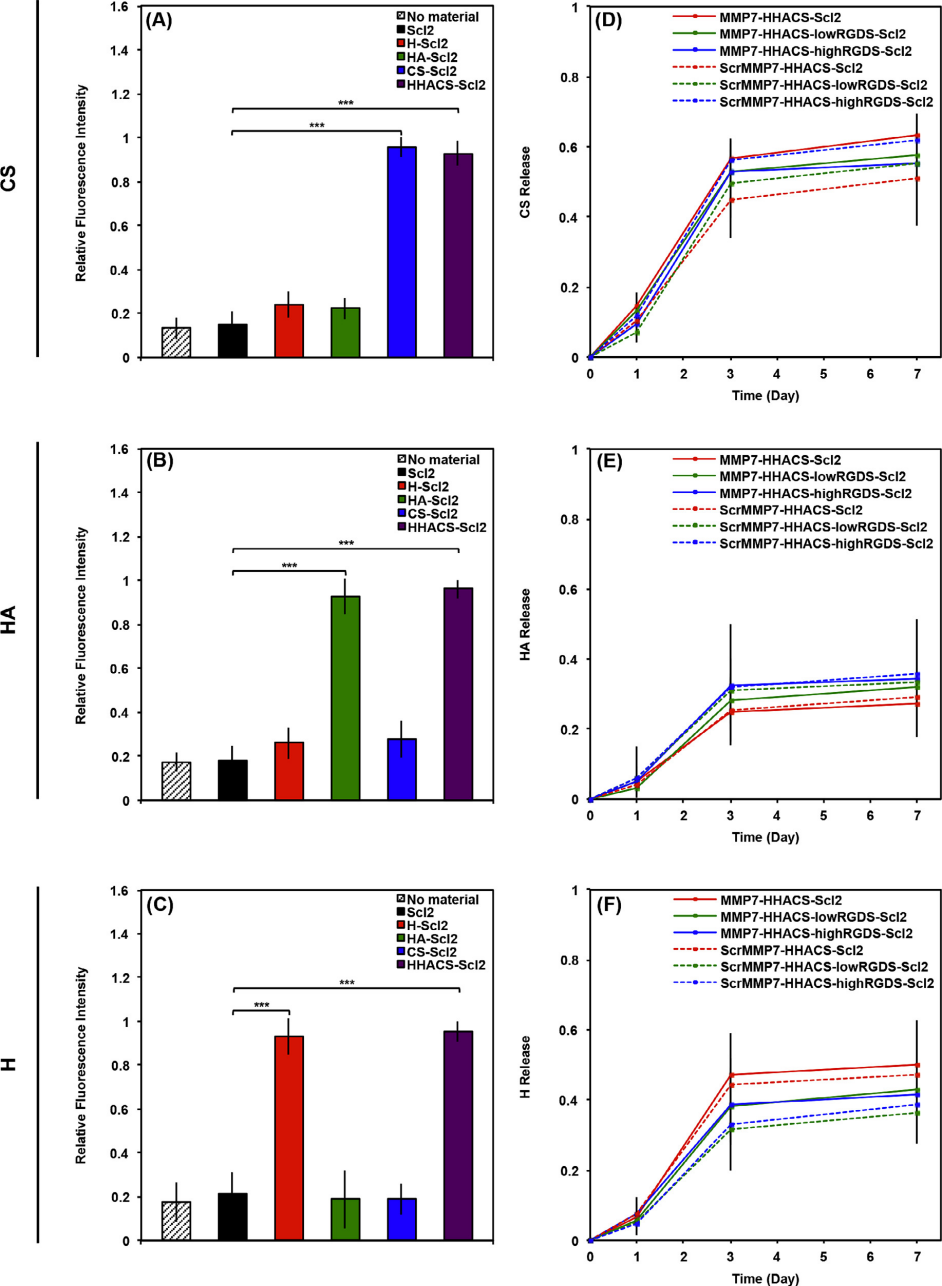
GAG binding on Scl2 proteins/hydrogels. (A–C) Binding of fluorescently-labeled (A) CS, (B) HA, and (C) heparin to Scl2 proteins. Empty wells were used as negative control denoted ‘no material’. (D–F) Release of fluorescently-labeled (D) CS, (E) HA, and (F) heparin from Scl2 hydrogels over 1 week. The fluorescence was measured in arbitrary units and relative binding of CS, HA, or heparin was normalized to the highest level of fluorescent intensity at the first time point to provide the fraction of CS, HA, or heparin released over time. Values represent means ± SD. ^***^p < 0.001 (n = 3).

Specific binding of CS, HA, and heparin to Scl2 functionalized with only one of the CS-, HA-, and heparin-binding peptide sequences was confirmed by incubating the samples with FITC-labeled GAGs (Fig.2A–C). Some non-specific binding of the GAGs to the non-binding Scl2 proteins was observed; however, this was not statistically significant compared to the blank, unmodified Scl2 protein. Furthermore, comparable release profiles of the GAGs from all hydrogels were observed (Fig.2D–F), possibly because the nature of the cross-linking peptides did not affect the binding or release of CS, HA, and heparin as the engineered binding sequences in the Scl2 backbone were the important elements here. Further, the dynamic microenvironment *in vivo* would likely result in noticeably different binding and release of the GAGs from the hydrogels.

#### 3.1.3 Morphological and mechanical characterization of hydrogels

**Fig. 3:**
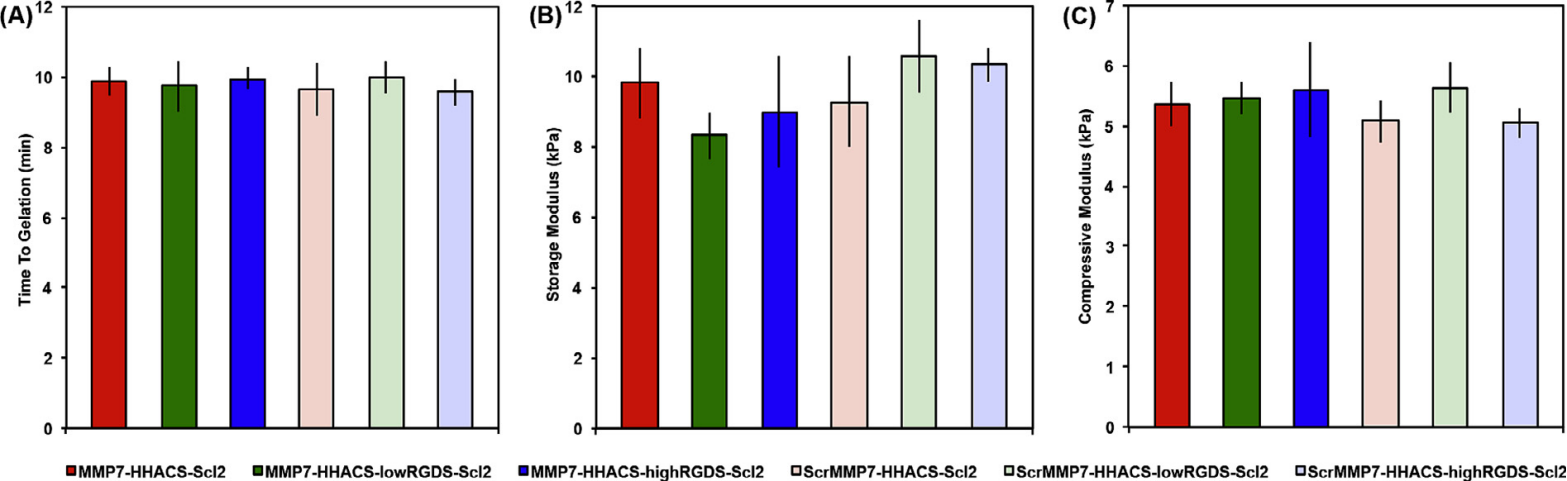
Mechanical properties of Scl2 hydrogels. (A) Time to gelation at a temperature of 37 °C, angular frequency of 6.28 rad/s, and strain of 0.5%. (B) Storage modulus determined from strain sweep up to 1% strain at a temperature of 37 °C and an angular frequency of 6.28 rad/s. (C) Dynamic mechanical analysis (DMA) used to determine the elastic modulus of compression of hydrogels compressed to 10% strain at 0.5% strain/min and 1 Hz. Values represent means ± SD (n = 3).

No differences in morphology were observed for the different hydrogel formulations using MP-SHG imaging (Fig. S5). As expected, there are no major observable differences in the macroscopic appearance of the hydrogel constructs without hMSCs compared to those with hMSCs (Fig. S6). The theoretical total degree of cross-linking induced by the MMP7 and ScrMMP7 peptides was kept constant for all hydrogel formulations to maintain comparable mechanical behavior, as hydrogel stiffness has been shown to affect cellular behavior such as adhesion, proliferation, and differentiation [41]. Oscillatory shear rheology was employed to confirm hydrogel formation and determine the time to gelation for all hydrogels (Figs. 3 and S7). All hydrogels formed after similar times and displayed comparable storage moduli. The equilibrium storage moduli of all hydrogel formulations remained in the linear elastic region up to 10% strain, after which point they decreased noticeably to failure point. Further mechanical characterization of the hydrogels using unconfined DMA confirmed these observations and the compression moduli were found to be ∼5 kPa at 1 Hz, with no statistical differences between the different hydrogel formulations. The failure strains of the hydrogels were comparable to other hydrogel-based systems commonly used in cartilage tissue engineering for treating focal cartilage defects [42], [43]. As the different hydrogels exhibited comparable morphological and mechanical characteristics, we were able to compare the effect of the enzymatically released and non-released RGDS peptide on cell behavior, decoupled from the mechanical properties and morphologies of the hydrogels.

### 3.2 Degradation kinetics of acellular hydrogels and RGDS peptide release

**Fig. 4:**
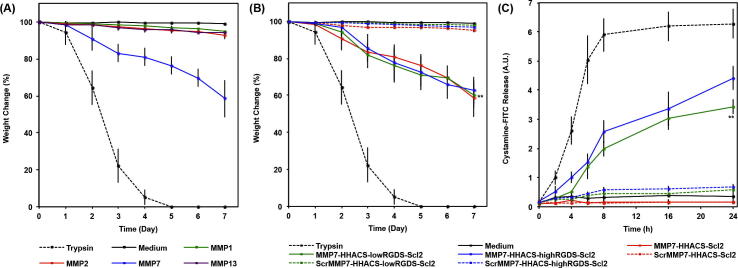
Degradation profile of acellular Scl2 hydrogels. (A) Degradation of hydrogels cross-linked via the MMP7-cleavable peptide and exposed to 30 ng/mL recombinant human MMP1, 2, 7, or 13 over time characterized as dry weight loss and expressed as a percentage of initial dry weight. (B) Degradation of hydrogels cross-linked via the MMP7-cleavable or ScrMMP7 peptides and exposed to 30 ng/mL recombinant human MMP7 over time characterized as dry weight loss and expressed as a percentage of initial dry weight. (C) Release of cystamine-FITC from hydrogels cross-linked via the MMP7-cleavable or ScrMMP7 peptides and exposed to 0.2 μg/mL recombinant human MMP7 over time characterized as fluorescent signal output. Trypsin-driven degradation is used as a positive control. Chondrogenic medium without exogenous enzymes is used as a negative control. Values represent means ± SD. ^**^p < 0.01 versus ScrMMP7-HHACS-Scl2 (n = 3).

Fig. 4 shows the degradation profiles of the acellular hydrogels when incubated with recombinant human MMP7, MMP1, MMP2, MMP13, or trypsin. As anticipated, all hydrogels cross-linked via the MMP7-cleavable peptide degraded considerably faster when exposed to MMP7 compared to MMP1, MMP2, and MMP13, for which minimal degradation was observed (Fig.4A). Previous studies have confirmed specific cleavage of the MMP7-cleavable peptide (PLELRA) in the presence of MMP7 [14], [21]. A small degree of non-specific degradation of the hydrogels by MMP1, MMP2, and MMP13 was observed likely due to the promiscuous cleavage activity of MMPs [20]. No statistical differences in hydrogel degradation kinetics were observed based on the concentration of RGDS peptide sequence, illustrating that MMP7 activity is not influenced by the presence of this peptide sequence (Fig.4B). As expected, hydrogels cross-linked via the non-cleavable ScrMMP7 peptide displayed minimal degradation in the presence of MMP7, validating the ScrMMP7 peptide sequence as a non-cleavable control in agreement with previous data [21].

FITC labeled cystamine was used as a model substrate in place of the RGDS peptide, in order to evaluate peptide release from the hydrogels over time after incubation with MMP7. All hydrogels cross-linked via the MMP7-cleavable peptide displayed a significantly higher release of cystamine-FITC from the hydrogels in the presence of MMP7. This suggests the RGDS peptide will be similarly released from these hydrogels as a result of MMP7 activity, compared to the non-degradable hydrogels that exhibited basal levels of cystamine-FITC release (Fig.4C). As per the experimental design of the degradable hydrogels, not all of the RGDS peptide will have the potential to be released within the experimental time frame of 24 h. Our studies demonstrated that after 24 h incubation of the MMP7-HHACS-lowRGDS-Scl2 and MMP7-HHACS-highRGDS-Scl2 hydrogels with exogenous MMP7, ∼55% and ∼70% of the RGDS peptide has the potential to be released compared to incubation with trypsin, respectively. However, it is likely that longer exposure to or higher concentrations of MMP7 would completely degrade the MMP7-cleavable hydrogels, thus fully releasing the RGDS peptide.

### 3.3 hMSC behavior in bioactive Scl2 hydrogels

#### 3.3.1 Cell viability and metabolic activity

**Fig. 5:**
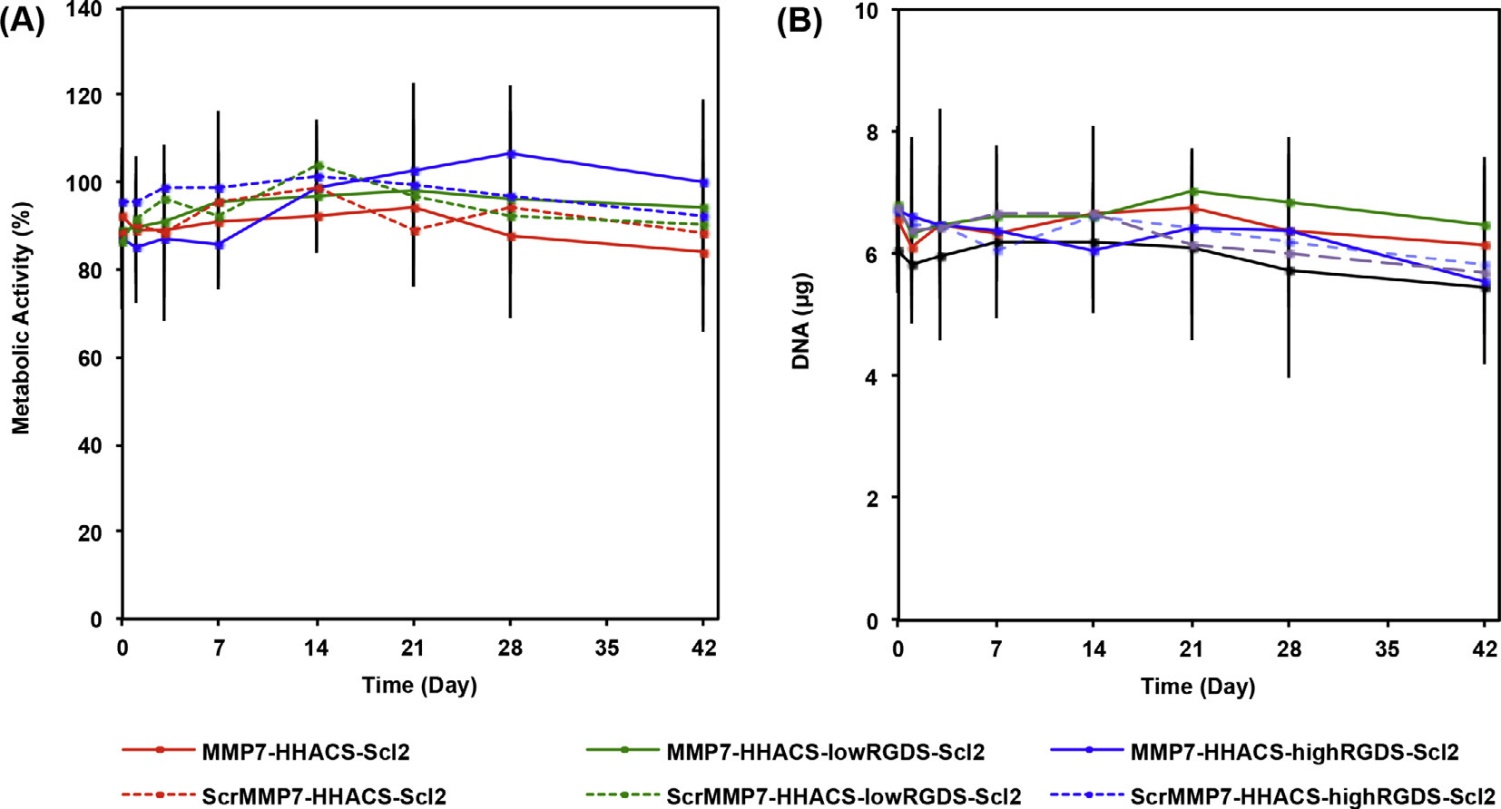
Metabolic activity and DNA content of hMSCs cultured in Scl2 hydrogels. (A) hMSC metabolic activity in hydrogels over 6 weeks in culture. (B) DNA content of hMSCs per construct in hydrogels cultured over 6 weeks in culture. All data normalized to day 0. Values represent means ± SD (n = 3 for each donor; N = 3 different bone marrow-derived hMSC donors).

hMSC viability in all hydrogels was qualitatively confirmed via a LIVE/DEAD® assay at day 42 and remained high for all hydrogels (Fig. S8). The metabolic activity of hMSCs and DNA content also remained elevated in all hydrogels throughout the culture period as determined by AlamarBlue® and PicoGreen® assays, respectively (Fig. 5). These results were expected due to the presence of the heparin-, HA-, and CS-binding peptides that have the ability to retain the cell-synthesized GAGs, and provide an environment suitable for cell viability. These matrix components have previously been shown to improve cell viability in hydrogels and are known to play important roles in a variety of cell–cell, cell–ECM, and protein interactions [36], [37]. The RGDS motif is widely used for aiding cell adhesion, proliferation, and maintaining cell viability in hydrogels [18].

#### 3.3.2 *In vitro* chondrogenesis

**Fig. 6:**
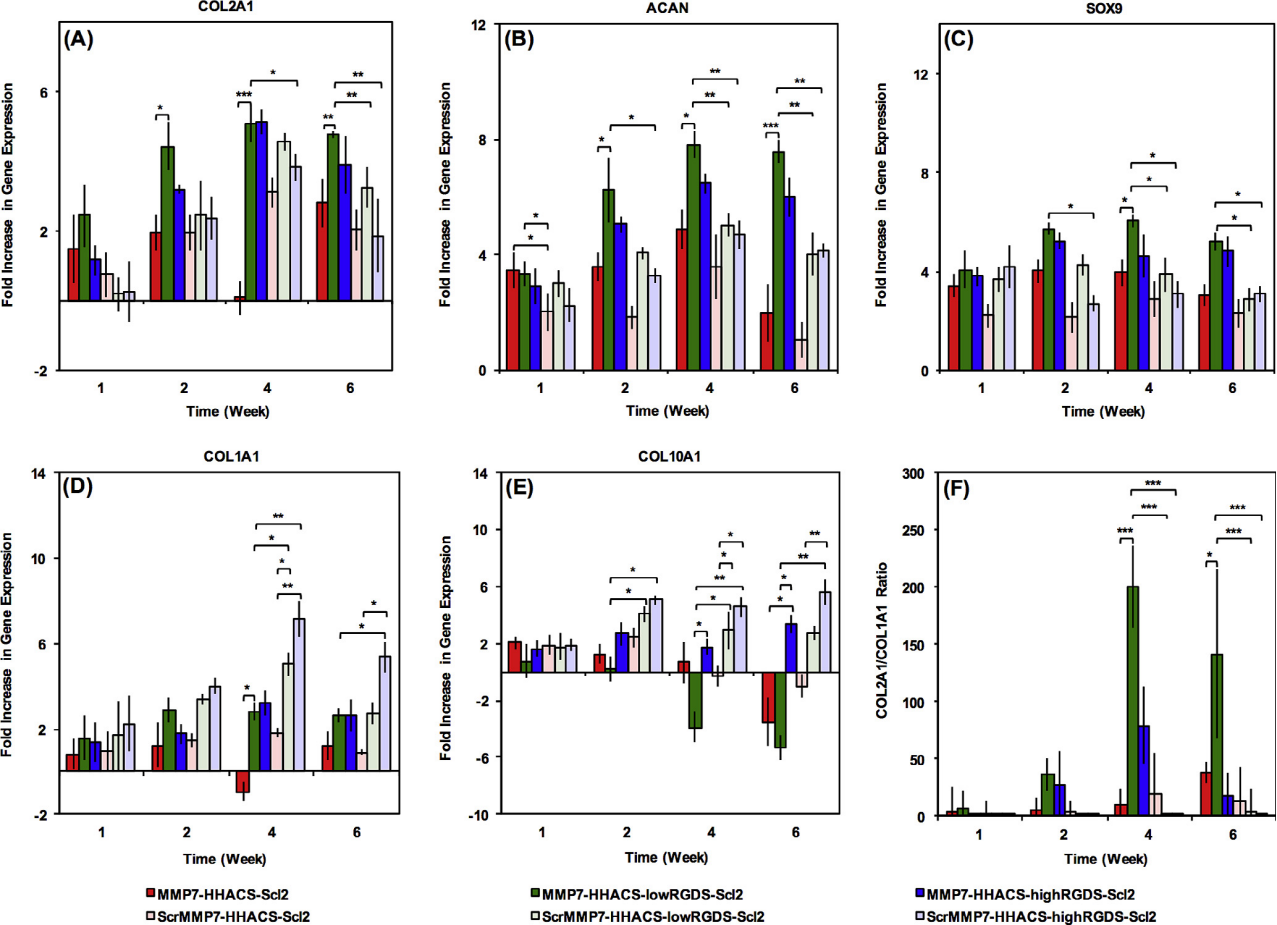
hMSC gene expression in Scl2 hydrogels. (A) COL2A1, (B) ACAN, (C) SOX9, (D) COL1A1, and (E) COL10A1 gene expression of hMSCs encapsulated in hydrogels over 6 weeks in culture, as analyzed using the ΔΔCt method. Data presented as a fold difference relative to undifferentiated hMSCs (calibrator) prior to encapsulation and normalized to GAPDH (housekeeping gene). (F) COL2A1/COL1A1 gene expression ratio. Values represent means ± SD. ^*^p < 0.05, ^**^p < 0.01, ^***^p < 0.001 (n = 3 for each donor; N = 3 different bone marrow-derived hMSC donors).

The chondrogenic differentiation of hMSCs encapsulated within MMP7-cleavable and non-cleavable ScrMMP7 hydrogels, with or without conjugated RGDS peptide, was evaluated to allow decoupling of the effect of the degradable hydrogels from the effect of RGDS peptide release. In effect, the presence of the MMP7-cleavable moieties in the hydrogels and the action of encapsulated cells via the release of MMP7 enables the release of RGDS peptide via enzymatic action (some directly from the cleavage and the rest via the release of Scl2 molecules with bound RGDS peptide), in contrast to that functionalized in non-cleavable ScrMMP7 hydrogels. The gene expression of encapsulated hMSC chondrogenic markers (COL2A1, ACAN, and SOX9) (Fig.6A–C) in the MMP7-HHACS-lowRGDS-Scl2 hydrogels were significantly up-regulated at multiple time points throughout the culture period, when compared to MMP7-HHACS-Scl2 (without RGDS peptides) and non-degradable hydrogels for which no or very low RGDS peptide release is expected throughout the culture period. Specifically, hMSCs in the MMP7-HHACS-lowRGDS-Scl2 hydrogels had significantly greater gene expression levels of all 3 chondrogenic markers at week 4 and week 6 (later time points) compared to all non-degradable hydrogels. This is in accordance with prior literature, which suggests that RGDS binding is beneficial in the early stages of chondrogenesis but can exhibit negative effects at later stages [18]. The gene expression profiles for COL1A1, a fibrocartilaginous tissue marker, and COL10A1, a hypertophic cartilage marker (Fig.6D–E), were the lowest for hMSCs encapsulated within the MMP7-HHACS-lowRGDS-Scl2 hydrogels compared to all non-degradable hydrogels for which they were up-regulated or remained at high levels at weeks 2–6. Delaying or inhibiting terminal chondrocyte differentiation of hMSCs toward fibrocartilaginous and/or hypertrophic phenotypes remains a critical challenge for cartilage tissue engineering strategies based on MSCs *in vivo* since this can result in the formation of repair tissue with inferior mechanical properties compared to native cartilage and/or mineralization of the newly formed tissue [44], [45], [46], [47], [48]. Our hydrogel constructs exhibited clear decreases in collagen type I and X levels over a period of several weeks compared to other similar systems [49], [50], [51], [52]. These findings were further supported by the COL2A1/COL1A1 gene expression ratio on the MMP7-HHACS-lowRGDS-Scl2 hydrogels, which remained high compared to all other hydrogel formulations throughout culture (Fig.6F). Taken together, these results suggest that the presence of the RGDS peptide is important for hMSC chondrogenesis during early time points, but its continued presence appears to have a negative impact on hMSC chondrogenesis. These results build on previously reported findings on the effect of transient exposure to RGD peptides on chondrogenesis by providing additional characterization of the cellular responses (notably with regards to the fibrocartilagenous and hypertrophic responses) in a different system [18], [24].

Interestingly, hMSCs in the MMP7-HHACS-lowRGDS-Scl2 hydrogels exhibited increased gene expression levels of chondrogenic and decreased expression of hypertrophic markers compared to those in the MMP7-HHACS-highRGDS-Scl2 hydrogels, with some genes/time points presenting statistically significant differences. This suggests that there may be an optimal amount of RGDS peptide required to promote hMSC chondrogenesis in the degradable hydrogels described in this study. Additionally, more extensive chondrogenic differentiation of hMSCs appears to be occurring in all degradable hydrogels compared to non-degradable hydrogels, which is likely due to less interference with matrix deposition resulting in greater neocartilaginous tissue accumulation and elaboration [14].

**Fig. 7:**
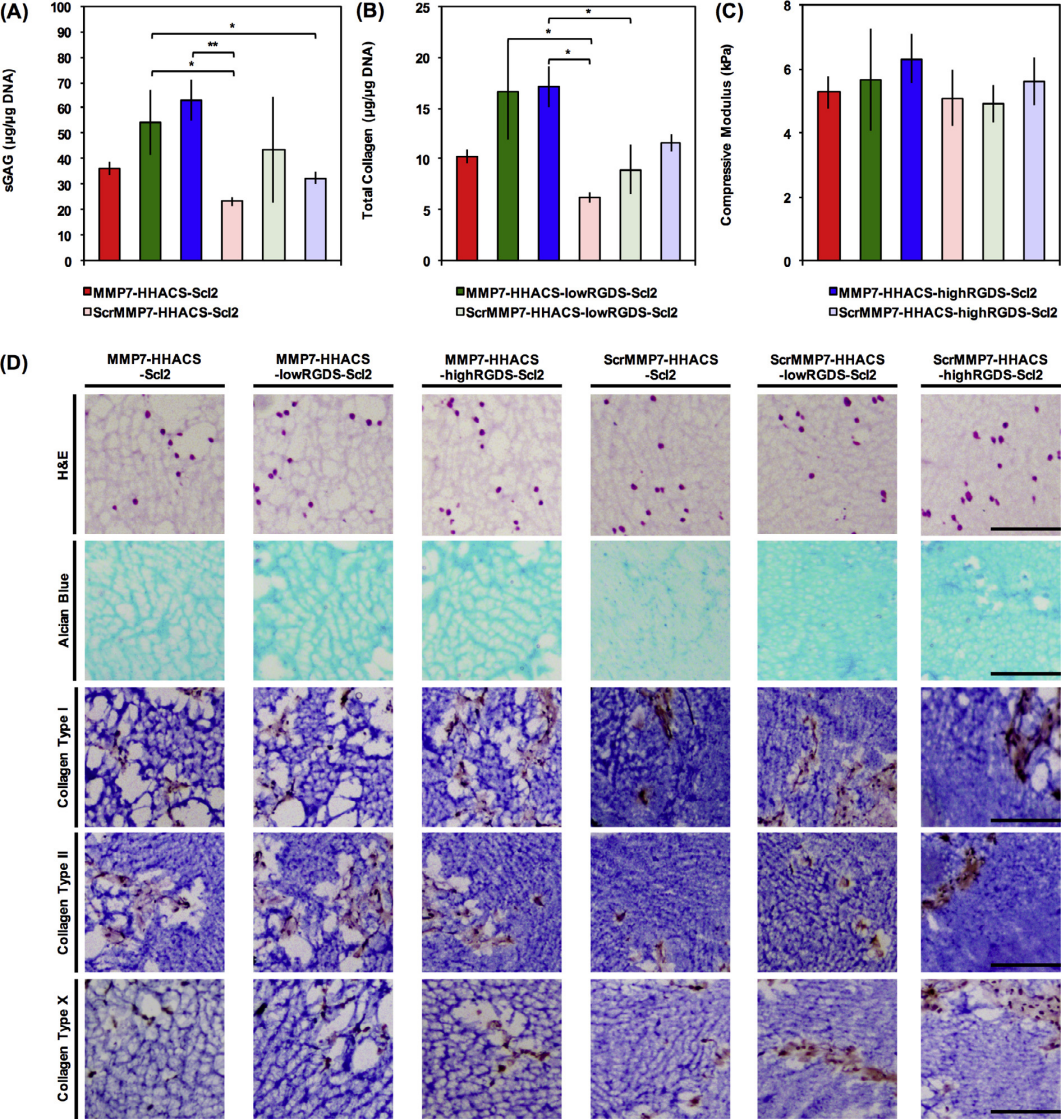
ECM accumulation and compressive modulus of hMSC-seeded Scl2 hydrogels. (A) Sulfated glycosaminoglycan (sGAG) content of tissue deposited by hMSCs in hydrogels over 6 weeks in culture. (B) Hydroxyproline content of tissue deposited by hMSCs in hydrogels over 6 weeks in culture as an estimation of total collagen content. (C) Dynamic mechanical analysis (DMA) used to determine the elastic modulus of compression of hMSC-seeded hydrogels compressed to 10% strain at 0.5% strain/min and 1 Hz after 6 weeks in culture. Values represent means ± SD. ^*^p < 0.05, ^**^p < 0.01, ^***^p < 0.001 (n = 3 for each donor; N = 3 different bone marrow-derived hMSC donors). (D) Representative histological and immunohistochemical examination of hMSC-seeded hydrogels after 6 weeks in culture. Hydrogels are stained with Haematoxylin and Eosin (H&E), Alcian Blue for sGAG, and by immunohistochemistry for collagen type I, collagen type II, and collagen type X, respectively, from top to bottom. Scale bars are 50 μm.

These findings at the gene expression level were less pronounced at the protein level after 6 weeks of culture. Nevertheless, the amount of sGAG matrix normalized to DNA content (Fig.7A) was greater for the MMP7-HHACS-lowRGDS-Scl2 hydrogels compared to the non-degradable hydrogels. A similar trend was observed for total collagen content normalized to DNA (Fig.7B), where the total collagen content in the MMP7-HHACS-lowRGDS-Scl2 hydrogels was higher than all non-degradable hydrogels. These results provided additional validity to the importance of the early presence of the RGDS peptide, followed by release from the hydrogels over time, for inducing hMSC chondrogenesis. These results also support the use of degradable hydrogel systems, with the hydrogel matrix being broken down and simultaneously replaced by neocartilaginous tissue, resulting in less interference during ECM elaboration. No statistical differences in compression moduli were observed between all hydrogel formulations (Figs. 7C and S9). This is likely in part because the degradable hydrogels were replaced by significantly higher amounts of cell-secreted ECM compared to the non-degradable hydrogels. The non-degradable hydrogels that exhibited minimal degradation accumulated significantly less ECM, resulting in their compression moduli not being significantly higher than the degradable hydrogels.

Histological examination of the hydrogels indicated a homogeneous distribution of cells and newly deposited matrix for all hydrogels at day 42 (Figs. 7D and S10). All hydrogels displayed high amounts of sGAG accumulation, with the exception of the ScrMMP7-HHACS-Scl2 hydrogels, where less sGAG matrix was evident. These results further support the sGAG accumulation data. Immunohistochemical evaluation of collagen type I, collagen type II, and collagen type X supported the gene expression results for all hydrogels. Staining for collagen type II was noticeably higher for the MMP7-HHACS-lowRGDS-Scl2 hydrogels compared to the ScrMMP7-HHACS-Scl2 hydrogels, while collagens types I and X were decreased. Evidently, there are more open spaces in the degradable hydrogels compared to the non-degradable hydrogels, which is likely due to a combination of hydrogel degradation and matrix accumulation and with additional time or *in vivo* incubation, these voids will likely be completely occupied with ECM.

#### 3.3.3 hMSC-mediated RGDS release

**Fig. 8:**
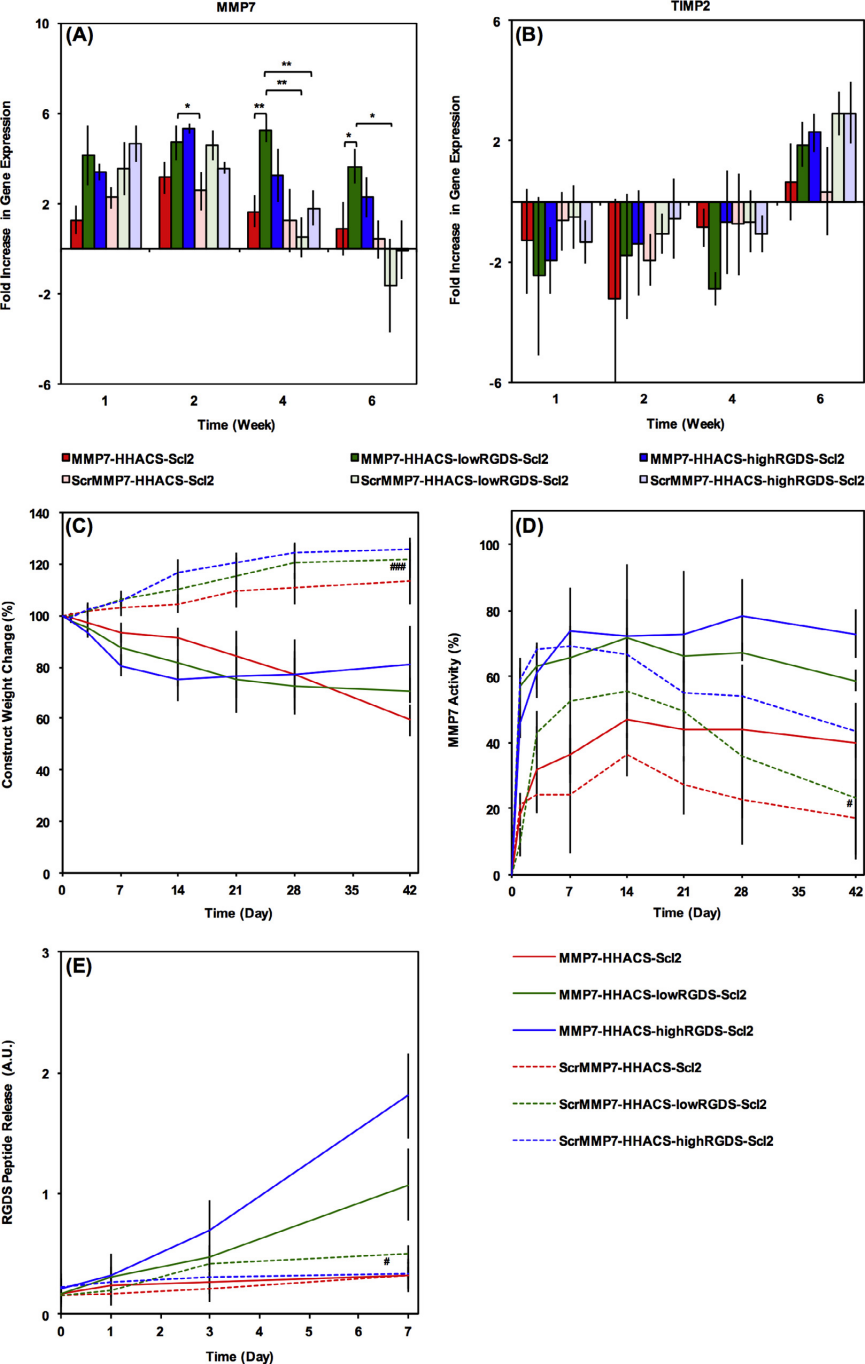
hMSC gene expression, construct weight change, enzymatic activity, and RGDS peptide release in Scl2 hydrogels. (A) MMP7 and (B) TIMP2 gene expression of hMSCs encapsulated in hydrogels over 6 weeks in culture, as analyzed using the ΔΔCt method. Data presented as a fold difference relative to undifferentiated hMSCs (calibrator) prior to encapsulation and normalized to GAPDH (housekeeping gene). (C) Scl2 hydrogel dry weight change over time in culture with hMSCs. Weight change was normalized to dry weight at day 0. (D) Activity of MMP7 in hydrogels over 6 weeks in culture. MMP7 activity was normalized to fluorescence signal output at day 0. (E) Accumulative release of cystamine-FITC, as an indicator of the RGDS peptide release from hMSC-seeded hydrogels cross-linked via the MMP7-cleavable or ScrMMP7 peptides over time characterized as fluorescent signal output. Values represent means ± SD. ^*^p < 0.05, ^#^*p* < 0.05 versus MMP7-HHACS-lowRGDS-Scl2, ^**^p < 0.01, ^***^p < 0.001, ^###^*p* < 0.001 versus MMP7-HHACS-lowRGDS-Scl2 (n = 3 for each donor; N = 3 different bone marrow-derived hMSC donors).

As the RGDS peptide had a significant effect on hMSC chondrogenesis, we anticipated to observe an effect on MMP7 gene expression and activity, a protease that is up-regulated in the early stages of chondrogenesis and down-regulated in the later stages. MMP7 gene expression levels were significantly up-regulated in the MMP7-HHACS-lowRGDS-Scl2 hydrogels compared to all other hydrogel formulations at week 6 (Fig.8A). In contrast, MMP7 gene expression levels by hMSCs encapsulated within the ScrMMP7-HHACS-Scl2 hydrogels were the lowest compared to all other hydrogels. The TIMP2 gene expression levels by hMSCs (Fig.8B), a known tissue inhibitor of MMP7 [20], did not exhibit significant changes between hydrogels.

While non-degradable hydrogels showed increases in weight over time, likely as a result of ECM accumulation without material degradation, MMP7-cleavable hydrogels exhibited decreased weight over time despite higher ECM accumulation as the hydrogel degraded simultaneously with the accumulation of ECM (Fig.8C). Furthermore, MMP7 activity was up-regulated for all hydrogel constructs, particularly for the MMP7-HHACS-lowRGDS-Scl2 and MMP7-HHACS-highRGDS-Scl2 hydrogels (Fig.8D). Additionally, hMSC-seeded hydrogels cross-linked via the MMP7-cleavable peptide demonstrated a significant release of cystamine-FITC, confirming the release of the RGDS peptide during culture, likely caused by endogenous MMP7 secreted by hMSCs undergoing chondrogenic differentiation (Fig.8E).

The temporal presentation of controlled concentrations of bioactive cues to cells in complex 3D scaffold systems holds great promise for recapitulating the intricate and dynamic microenvironment of the native tissue [53], [54], [55], [56]. In mimicking the native pericellular microenvironment, it is of great importance to modulate the temporal activation of cell-signaling pathways to avoid improper cell differentiation and function. Our findings suggest the need to incorporate spatial complexity within such 3D tissue-engineered systems to further recapitulate aspects of the native tissue environment. Additionally, more than one spatiotemporally presented bioactive signal may be required to regenerate zonal organization of tissues such as articular cartilage that is deemed important for its multiple functions [57], [58], [59]. As such, the modularity of our hydrogel system allows for greater control over specific cellular processes, with potential benefits for clinical translation. Further, our novel hydrogel paltform can be easily adapted for numerous other regenerative medicine and tissue engineering applications through a careful selection of tethered and backbone modified bioactive cues.

## 4 Conclusions

In this work, we have developed novel hydrogels based on collagen-mimetic proteins with cell-mediated control over the temporal presentation of bioactive cues, in this case the RGDS peptide. The enzymatic release of tethered RGDS from a hydrogel that also incorporated peptide sequences to dynamically, specifically, and non-covalently bind heparin, HA, and CS was shown to significantly promote hMSC chondrogenesis compared to hydrogels for which the RGDS peptide remained tethered to the backbone throughout the culture period. Additionally, hydrogels functionalized with a low concentration of the RGDS peptide resulted in decreased collagen type I and X expression. The results in this work highlight the importance of the temporal presentation of bioactive cues and present an approach to achieve this degree of complexity. This work presents an additional example of the architectural flexibility and modularity of our novel collagen-mimetic hydrogel platform and its potential utility in tissue engineering and regenerative medicine applications.
