# Supplementary material for: Enhanced articular cartilage by human mesenchymal stem cells in enzymatically mediated transiently RGDS-functionalized collagen-mimetic hydrogels
Source: Acta Biomater. 2017 Mar 15;51:75–88. doi: 10.1016/j.actbio.2017.01.028 (PMC5360098; doi:10.1016/j.actbio.2017.01.028)
Supplement: Supplementary data 1 [file mmc1.pdf]

Supplementary Information

Enhanced Articular Cartilage by Human Mesenchymal Stem Cells in Enzymatically Mediated Transiently RGDS–Functionalized Collagen–Mimetic Hydrogels

Paresh A. Parmar, Jean–Philippe St–Pierre, Lesley W. Chow, Christopher D. Spicer, Violet Stoichevska, Yong Y. Peng, Jerome A. Werkmeister, John A.M. Ramshaw, and Molly M. Stevens

Synthesis and purification of MMP7–cleavable, non–cleavable ScrMMP7, and cyclic RGDS peptides.

The MMP7 (PLELRA)–cleavable and non–cleavable scrambled MMP7 (ScrMMP7; PALLRE) peptides were synthesized manually on a 2 mmol scale using standard Fmoc solid phase peptide synthesis techniques as previously described [34]. For each coupling, the Fmoc protecting group was removed with 20% (v/v) piperidine in DMF followed by washing with DCM and DMF. Amino acids were activated by adding 4 molar equivalent of each Fmoc protected amino acid to 3.95 molar equivalent of HBTU and dissolved in DMF. Six molar equivalent of DIEA was added to the amino acid solution and the coupling solution added to the resin. The coupling reaction was allowed to proceed for 120–180 min before the resin was washed in DCM and DMF. Ninhydrin tests were performed after each Fmoc deprotection and coupling step to monitor the presence of free amines.

Two Fmoc–Lys(Mtt)–OH were also coupled to the peptides at the N– and C– termini each using methods described above. The ninhydrin test for free amines was conducted at room temperature for 30 min since the Mtt protecting group is heat–labile. The Mtt protecting group was removed from the amine on the lysine side chain with 5% (v/v) TFA in DCM for 2 min on the shaker. The solution turns bright yellow as the Mtt is removed, and this was repeated at least 5 times or until the solution was clear. The beads were washed thoroughly with DCM and DMF before running the ninhydrin test at room temperature for 30 min. To attach acrylate groups to the peptide, acryloyl chloride was activated by adding 10 molar

equivalents of acryloyl chloride and 10 molar equivalents of DIEA in DCM. The coupling was verified with the ninhydrin test at room temperature for 30 min.

Once the synthesis was completed, the peptides were cleaved in 95% (v/v) trifluoroacetic acid (TFA), 2.5% (v/v) triisopropyl silane (TIS), and 2.5% (v/v) H_2O for 240 min. TFA was removed using rotary evaporation, and the peptide residues were precipitated and washed with cold diethyl ether by centrifugation. The peptide precipitates were then allowed to dry under vacuum to remove residual ether. The peptides were purified (Fig. S1) using reversed phase preparative high performance liquid chromatography (HPLC; Shimadzu) in an acetonitrile/water gradient under acidic conditions on a Phenomenex C18 Gemini NX column (5 μm pore size, 110 Å particle size, 150 x 21.2 mm). Following purification, the peptides were lyophilized on a freeze dryer (Labconco) for storage prior to use. The purified peptide masses were verified by matrix assisted laser desorption spectroscopy (MALDI; Waters).

A cyclic RGDS peptide (GRGDSC) was synthesized at a 1 mmol scale on a 2–chlorotrityl chloride resin (100–200 mesh; VWR). Fmoc–Cys(Trt)–OH (Novabiochem) was dissolved at 1 molar equivalent in DCM with a small amount of DMF added until fully solubilized. Half the solution was added to the resin along with 500 μL of DIEA for 15 min shaking on a wrist action shaker. This was repeated with the remaining solution followed by thorough washing with DMF and DCM. A ninhydrin test was performed to monitor the coupling by detecting the presence of free amines. The remaining free amines were capped by adding a solution of 5% (v/v) acetic anhydride with 2.5% (v/v) DIEA in DMF for 10 min with shaking, and repeated with 5 min shaking using fresh solution. The resin was then washed thoroughly with DCM and DMF before the ninhydrin test. The Fmoc protecting group was removed as described above and Fmoc–Asp(Otbu)–Ser(psiMe,Mepro)–OH (Merck) was coupled at 2 molar equivalents with 1.95 molar equivalents of HBTU and 3 molar equivalents of DIEA in DMF. The remaining free amines were capped, and all other amino acids were coupled as described above. The protected peptide was cleaved from the resin by adding 10 mL of 5% (v/v) TFA in DCM for 10 min with shaking. The solution was drained into a round

bottom flask and the resin rinsed with DCM until the solution in the synthesis vessel was clear. The DCM and TFA were removed carefully by rotary evaporation, leaving approximately 40 mL of solution to avoid cleaving the protecting groups from the peptide. Ammonium hydroxide (10 mL) was added to neutralize the TFA followed by acetonitrile to increase peptide solubility. The protected peptide was purified by reverse phase preparative HPLC running a mobile phase gradient of 80% ultrapure H_2O and 20% (v/v) ACN to 100% (v/v) ACN with 0.1% (v/v) TFA. The solvent was removed by rotary evaporation until the protected peptide was completely dry, and then re–dissolved in DMF at 1 mg/mL. The peptide was cyclized by adding 2 equivalents of benzotriazol–1–yl–oxytripyrrolidinophosphonium hexafluorophosphate (PyBop; AGTC Bioproducts) and 3 equivalents of DIEA overnight. The DMF was removed by rotary evaporation and the remaining product was re–dissolved in acetonitrile/water until solubilized and purified by reverse phase preparative HPLC as described above. The remaining protecting groups were removed using 95% (v/v) TFA with 2.5% (v/v) H_2O and 2.5% (v/v) TIS. The peptide was precipitated in cold DEE and purified by reverse phase preparative HPLC. Liquid chromatography–mass spectrometry (LC–MS) was performed on an Agilent 6130 Quadrupole LC–MS coupled to an Agilent 1260 Infinity LC using a 150 x 4.6 mm Phenomenex Gemini NX C18 column with a 5 μm pore size and 100 Å particle size. Ultrapure H_2O and acetonitrile each containing 0.1% (v/v) formic acid (VWR) by volume were used for the mobile phase at a flow rate of 1 mL/min. The peptide was eluted with a gradient of 95% (v/v) H_2O to 95% (v/v) acetonitrile over 11 min. The electrospray source was operated with a capillary voltage of 3.2 kV and a cone voltage of 25 V with nitrogen used as the nebulizer and de–solvation gas at a total flow of 600 L/h. Although cyclic GRGDSC did not stick to the column, electrospray ionization (ESI) of an early elution confirmed the correct mass.

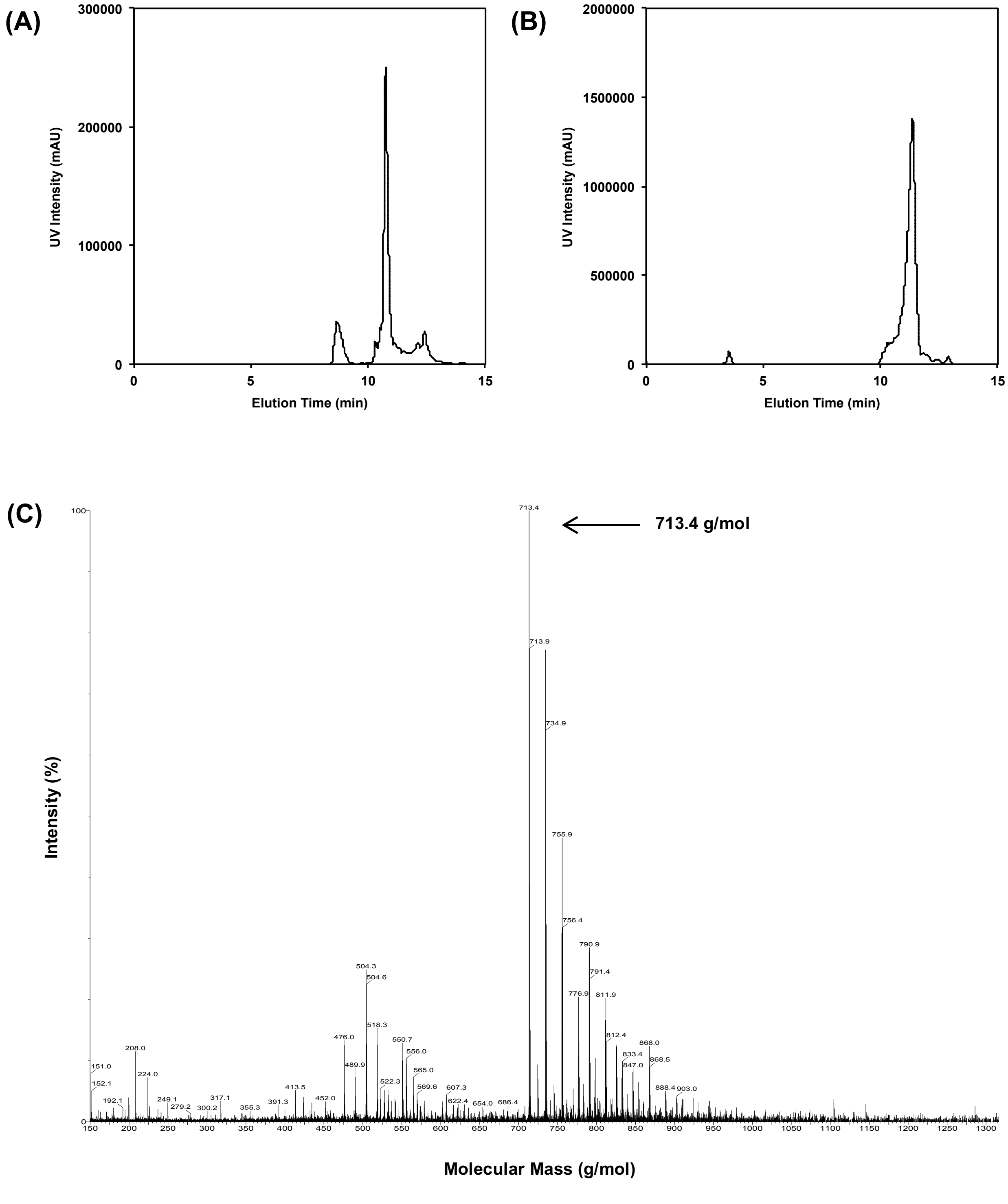

Fig. S1. Representative reversed phase high performance liquid chromatography (HPLC) chromatograms and electrospray ionization (ESI) spectra of (A, C) MMP7 (MW/2: 713.4 g/mol, MW: 1426.8 g/mol) and (B) ScrMMP7 (MW/2: 713.4 g/mol, MW: 1426.8 g/mol) peptides, respectively.

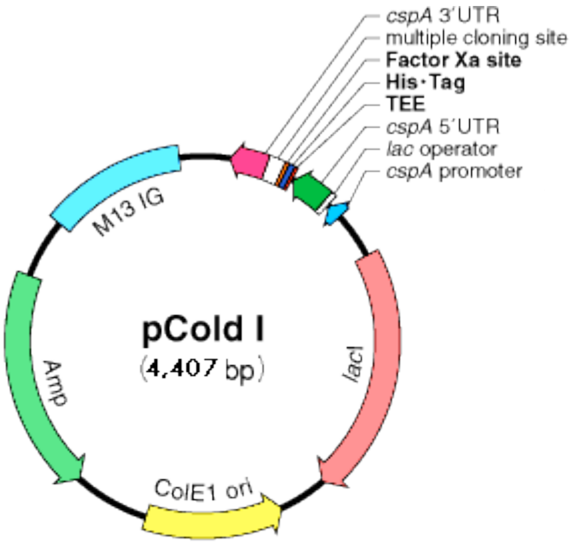

pCold I vector sequence:

```
    1 AAGGAATGGT GTGGCCGATT AATCATAAAT ATGAAAAATA ATTGTTGCAT CACCCGCCAA TGCGTGGCTT AATGCACATC AAATTGTGAG CGGATAACAA

                BmtI
              ------+
                NheI
              -+-----
  101 TTTGATGTGC TAGCGCATAT CCAGTGTAGT AAGGCAAGTC CCTTCAAGAG TTATCGTTGA TACCCCTCGT AGTGCACATT CCTTTAACGC TTCAAAATCT

  201 GTAAAGCACG CCATATCGCC GAAAGGCACA CTTAATTATT AAGAGGTAAT ACACCATGAA TCACAAAGTG CATCATCATC ATCATCATAT CGAAGGTAGG

                           AbsI
                        --+------
                    KpnI                                    PstI
                   -----+                                 ------+
              SacI        PspXI                      AccI
            ------+     --+------                   --+---
            Eco53kI        XhoI         EcoRI        SalI
            ---+---      -+-----       -+----       -+----
       NdeI        Acc65I        BamHI       HindIII     BspMI    XbaI
      --+---       -+----       -+----       -+-----    -------  -+----
  301 CATATGGAGC TCGGTACCCT CGAGGGATCC GAATTCAAGC TTGTCGACCT GCAGTCTAGA TAGGTAATCT CTGCTTAAAA GCACAGAATC TAAGATCCCT

                                                       ClaI
                                                      --+---
  401 GCCATTTGGC GGGGATTTTT TTATTTGTTT TCAGGAAATA AATAATCGAT CGCGTAATAA AATCTATTAT TATTTTTGTG AAGAATAAAT TTGGGTGCAA

          BsmI    Eco0109I                                               NspI
         ------   --+----                                               -----+
  501 TGAGAATGCG CAGGCCCTTT CGTCTCGCGC GTTTCGGTGA TGACGGTGAA AACCTCTGAC ACATGCAGCT CCCGGAGACG GTCACAGCTT GTCTGTAAGC

  601 GGATGCCGGG AGCAGACAAG CCCGTCAGGG CGCGTCAGCG GGTGTTGGCG GGTGTCGGGG CTGGCTTAAC TATGCGGCAT CAGAGCAGAT TGTACTGAGA

  701 GTGCACCATA AAATTGTAAA CGTTAATATT TTGTTAAAAT TCGCGTTAAA TTTTTGTTAA ATCAGCTCAT TTTTTAACCA ATAGGCCGAA ATCGGCAAAA

           PsiI
          ---+--
  801 TCCCTTATAA ATCAAAAGAA TAGCCCGAGA TAGGGTTGAG TGTTGTTCCA GTTTGGAACA AGAGTCCACT ATTAAAGAAC GTGGACTCCA ACGTCAAAGG

                                          BsaAI
                                         ---+--
  901 GCGAAAAACC GTCTATCAGG GCGATGGCCC ACTACGTGAA CCATCACCCA AATCAAGTTT TTTGGGGTCG AGGTGCCGTA AAGCACTAAA TCGGAACCCT

                                              NaeI
                                            ---+---
                                             NgoMIV
                                            -+-----
 1001 AAAGGGAGCC CCCGATTTAG AGCTTGACGG GGAAAGCCGG CGAACGTGGC GAGAAAGGAA GGGAAGAAAG CGAAAGGAGC GGGCGCTAGG GCGCTGGCAA

 1101 GTGTAGCGGT CACGCTGCGC GTAACCACCA CACCCGCCGC GCTTAATGCG CCGCTACAGG GCGCGTACTA TGGTTGCTTT GACGTATGCG GTGTGAAATA

 1201 CCGCACAGAT GCGTAAGGAG AAAATACCGC ATCAGGCGTC AGGTGGCACT TTTCGGGGAA ATGTGCGCGG AACCCCTATT TGTTTATTTT TCTAAATACA

 1301 TTCAAATATG TATCCGCTCA TGAGACAATA ACCCTGATAA ATGCTTCAAT AATATTGAAA AAGGAAGAGT ATGAGTATTC AACATTTCCG TGTCGCCCTT

 1401 ATTCCCTTTT TTGCGGCATT TTGCCTTCCT GTTTTTGCTC ACCCAGAAAC GCTGGTGAAA GTAAAAGATG CTGAAGATCA GTTGGGTGCA CGAGTGGGTT

                                                                    XmnI
                                                                -----+-----
 1501 ACATCGAACT GGATCTCAAC AGCGGTAAGA TCCTTGAGAG TTTTCGCCCC GAAGAACGTT TTCCAATGAT GAGCACTTTT AAAGTTCTGC TATGTGGCGC

                                                                                        ScaI
                                                                                       ---+--
 1601 GGTATTATCC CGTATTGACG CCGGGCAAGA GCAACTCGGT CGCCGCATAC ACTATTCTCA GAATGACTTG GTTGAGTACT CACCAGTCAC AGAAAAGCAT

 1701 CTTACGGATG GCATGACAGT AAGAGAATTA TGCAGTGCTG CCATAACCAT GAGTGATAAC ACTGCGGCCA ACTTACTTCT GACAACGATC GGAGGACCGA

 1801 AGGAGCTAAC CGCTTTTTTG CACAACATGG GGGATCATGT AACTCGCCTT GATCGTTGGG AACCGGAGCT GAATGAAGCC ATACCAAACG ACGAGCGTGA

 1901 CACCACGATG CCTGTAGCAA TGGCAACAAC GTTGCGCAAA CTATTAACTG GCGAACTACT TACTCTAGCT TCCCGGCAAC AATTAATAGA CTGGATGGAG
```

```
                                               BglI                                                   BsaI
                                           -------+----                                             -------
2001   GCGGATAAAG TTGCAGGACC ACTTCTGCGC TCGGCCCTTC CGGCTGGCTG GTTTATTGCT GATAAATCTG GAGCCGGTGA GCGTGGGTCT CGCGGTATCA

2101   TTGCAGCACT GGGGCCAGAT GGTAAGCCCT CCCGTATCGT AGTTATCTAC ACGACGGGGA GTCAGGCAAC TATGGATGAA CGAAATAGAC AGATCGCTGA

2201   GATAGGTGCC TCACTGATTA AGCATTGGTA ACTGTCAGAC CAAGTTTACT CATATATACT TTAGATTGAT TTAAAACTTC ATTTTTAATT TAAAAGGATC

2301   TAGGTGAAGA TCCTTTTTGA TAATCTCATG ACCAAAATCC CTTAACGTGA GTTTTCGTTC CACTGAGCGT CAGACCCCGT AGAAAAGATC AAAGGATCTT

2401   CTTGAGATCC TTTTTTTCTG CGCGTAATCT GCTGCTTGCA AACAAAAAAA CCACCGCTAC CAGCGGTGGT TTGTTTGCCG GATCAAGAGC TACCAACTCT

2501   TTTTCCGAAG GTAACTGGCT TCAGCAGAGC GCAGATACCA AATACTGTTC TTCTAGTGTA GCCGTAGTTA GGCCACCACT TCAAGAACTC TGTAGCACCG

                                           AlwNI
                                         ------+--
2601   CCTACATACC TCGCTCTGCT AATCCTGTTA CCAGTGGCTG CTGCCAGTGG CGATAAGTCG TGTCTTACCG GGTTGGACTC AAGACGATAG TTACCGGATA

2701   AGGCGCAGCG GTCGGGCTGA ACGGGGGGTT CGTGCACACA GCCCAGCTTG GAGCGAACGA CCTACACCGA ACTGAGATAC CTACAGCGTG AGCTATGAGA

2801   AAGCGCCACG CTTCCCGAAG GGAGAAAGGC GGACAGGTAT CCGGTAAGCG GCAGGGTCGG AACAGGAGAG CGCACGAGGG AGCTTCCAGG GGGAAACGCC

2901   TGGTATCTTT ATAGTCCTGT CGGGTTTCGC CACCTCTGAC TTGAGCGTCG ATTTTTGTGA TGCTCGTCAG GGGGGCGGAG CCTATGGAAA AACGCCAGCA

3001   ACGCGGCCTT TTTACGGTTC CTGGCCTTTT GCTGGCCTTT TGCTCACATA GTCATGCCCC GCGCCCACCG GAAGGAGCTG ACTGGGTTGA AGGCTCTCAA

3101   GGGCATCGGT CGAGATCCCG GTGCCTAATG AGTGAGCTAA CTTACATTAA TTGCGTTGCG CTCACTGCCC GCTTTCCAGT CGGGAAACCT GTCGTGCCAG

                                                              BbeI
                                                            ------+
                                                              SfoI
                                                            ----+--
                                                              NarI
                                                            ---+---
                                                              KasI
                                                            --+----
3201   CTGCATTAAT GAATCGGCCA ACGCGCGGGG AGAGGCGGTT TGCGTATTGG GCGCCAGGGT GGTTTTTCTT TTCACCAGTG AGACGGGCAA CAGCTGATTG

                                                                                                    HpaI
                                                                                                   ---+--
3301   CCCTTCACCG CCTGGCCCTG AGAGAGTTGC AGCAAGCGGT CCACGCTGGT TTGCCCCAGC AGGCGAAAAT CCTGTTTGAT GGTGGTTAAC GGCGGGATAT

                                                    EcoRV                                   BssHII
                                                   ---+--                                  -+-----
3401   AACATGAGCT GTCTTCGGTA TCGTCGTATC CCACTACCGA GATATCCGCA CCAACGCGCA GCCCGGACTC GGTAATGGCG CGCATTGCGC CCAGCGCCAT

3501   CTGATCGTTG GCAACCAGCA TCGCAGTGGG AACGATGCCC TCATTCAGCA TTTGCATGGT TTGTTGAAAA CCGGACATGG CACTCCAGTC GCCTTCCCGT

                                                                                                 ApaI
                                                                                                -----+
                                                                                                PspOMI
                                                                                                -+----
3601   TCCGCTATCG GCTGAATTTG ATTGCGAGTG AGATATTTAT GCCAGCCAGC CAGACGCAGA CGCGCCGAGA CAGAACTTAA TGGGCCCGCT AACAGCGCGA

              BstEII
             -+------
3701   TTTGCTGGTG ACCCAATGCG ACCAGATGCT CCACGCCCAG TCGCGTACCG TCTTCATGGG AGAAAATAAT ACTGTTGATG GGTGTCTGGT CAGAGACATC

                                                                                                        AflIII
                                                                                                       -+-----
                                                                                         BclI            MluI
                                                                                        -+----         -+-----
3801   AAGAAATAAC GCCGGAACAT TAGTGCAGGC AGCTTCCACA GCAATGGCAT CCTGGTCATC CAGCGGATAG TTAATGATCA GCCCACTGAC GCGTTGCGCG

3901   AGAAGATTGT GCACCGCCGC TTTACAGGCT TCGACGCCGC TTCGTTCTAC CATCGACACC ACCACGCTGG CACCCAGTTG ATCGGCGCGA GATTTAATCG

4001   CCGCGACAAT TTGCGACGGC GCGTGCAGGG CCAGACTGGA GGTGGCAACG CCAATCAGCA ACGACTGTTT GCCCGCCAGT TGTTGTGCCA CGCGGTTGGG

4101   AATGTAATTC AGCTCCGCCA TCGCCGCTTC CACTTTTTCC CGCGTTTTCG CAGAAACGTG GCTGGCCTGG TTCACCACGC GGGAAACGGT CTGATAAGAG

                BstAPI
             --------+---
4201   ACACCGGCAT ACTCTGCGAC ATCGTATAAC GTTACTGGTT TCACATTCAC CACCCTGAAT TGACTCTCTT CCGGGCGCTA TCATGCCATA CCGCGAAAGG

                                                                    EcoNI
                                                                -----+------
4301   TTTTGCGCCA TTCGATGGTG TCCGGGATCT CGACGCTCTC CCTTATGCGA CTCCTGCATT AGGAAGCAGC CCAGTAGTAG GTTGAGGCCG TTGAGCACCG

4401   CCGCCGC
```

Fig. S2. A fully annotated plasmid map of the pColdI vector system used to sub–clone the final DNA sequence for expression in *E. coli*.

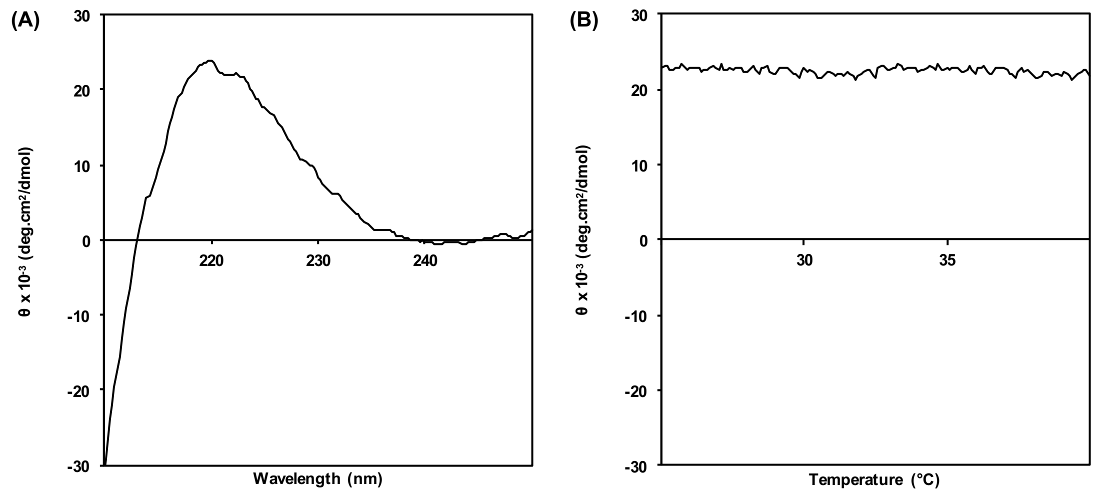

Fig. S3. Secondary structure of Scl2 proteins. (A) The ellipticity of the functionalized Scl2 proteins monitored from 210 to 250 nm at 37 °C. (B) The ellipticity of the functionalized Scl2 proteins monitored at 220 nm from 25 to 40 °C.

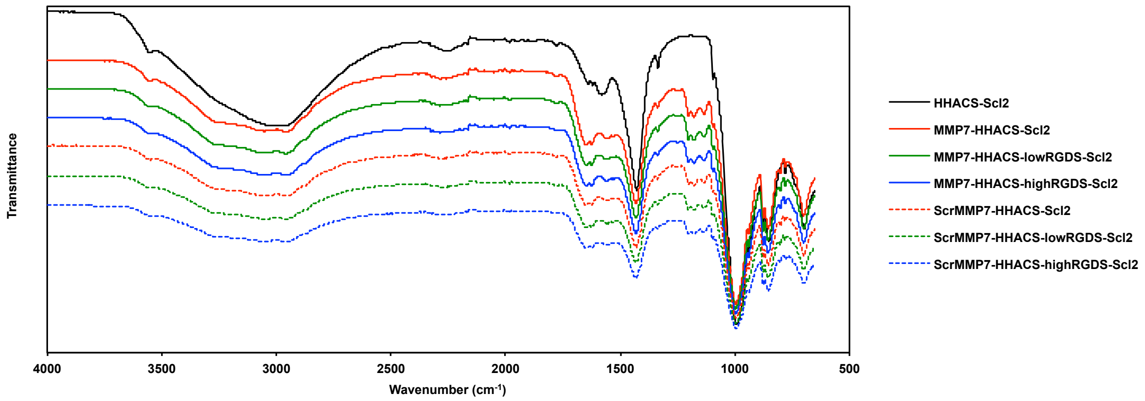

Fig. S4. Representative Fourier transform infrared (FTIR) spectra of functionalized Scl2 proteins confirming the conjugation of Scl2 with acrylate–functionalized MMP7–cleavable and non–cleavable ScrMMP7 peptides.

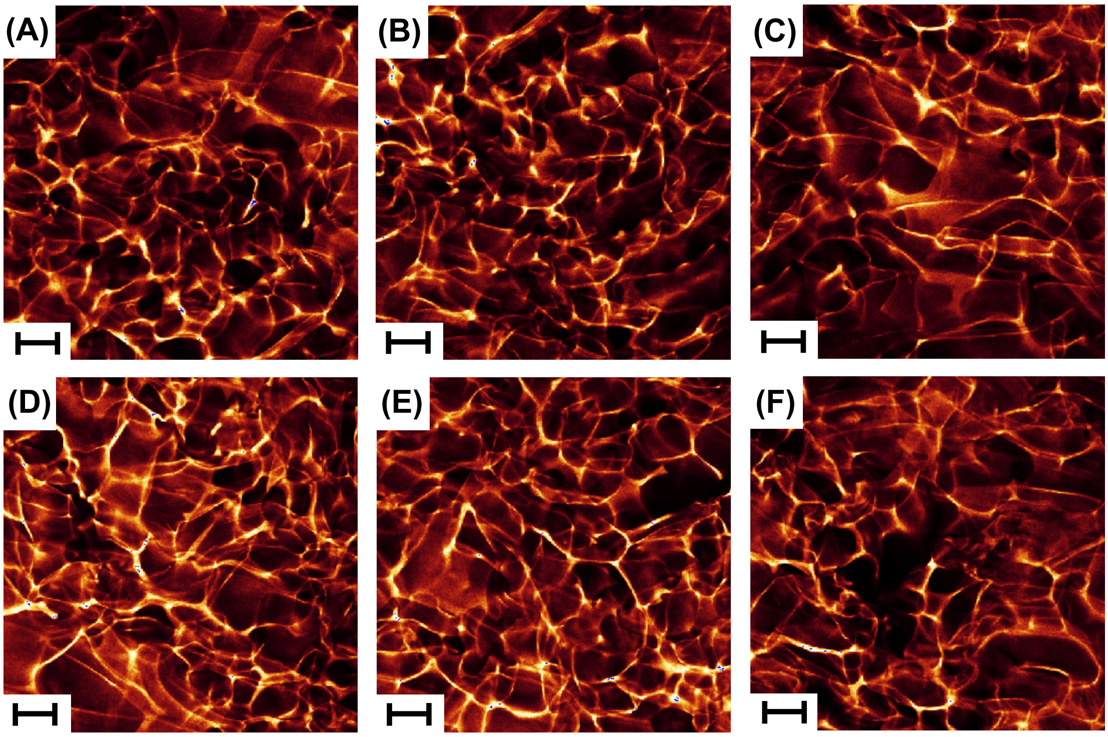

Fig. S5. Representative multi–photon second harmonic generation (MP–SHG) images of acellular (A) MMP7-HHACS-Scl2, (B) MMP7-HHACS-lowRGDS-Scl2, (C) MMP7-HHACS-highRGDS-Scl2, (D) ScrMMP7-HHACS-Scl2, (E) ScrMMP7-HHACS-lowRGDS-Scl2, and (F) ScrMMP7-HHACS-highRGDS-Scl2 hydrogels (scale bars are 10 μm).

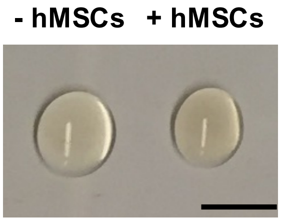

Fig. S6. Representative photos of acellular and hMSC–seeded MMP7-HHACS-Scl2 hydrogel constructs (scale bars are 1 cm).

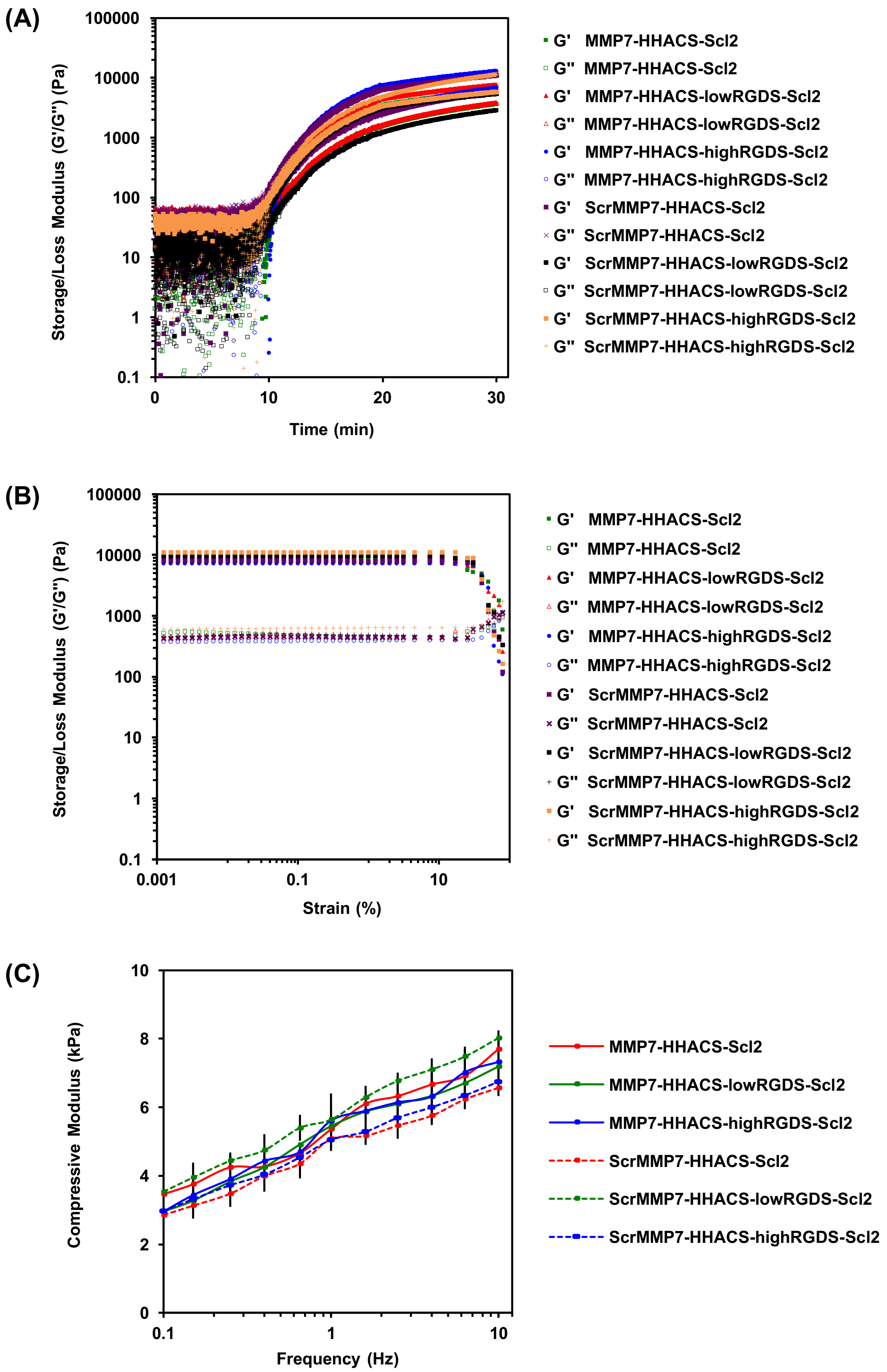

Fig. S7. Mechanical properties of acellular functionalized Scl2 hydrogels. (A) Time to gelation determined at a temperature of 37 °C, angular frequency of 6.28 rad/s, and strain of 0.5% shown as G’

and G’’. (B) Strain sweep at a temperature of 37 °C and an angular frequency of 6.28 rad/s shown as G’ and G’’. (C) Dynamic mechanical analysis (DMA) used to determine the elastic modulus in unconfined compression of hydrogels compressed to 10% strain at 0.5% strain/min from 0.1 to 10 Hz and (B). Values represent means ± SD (n = 3).

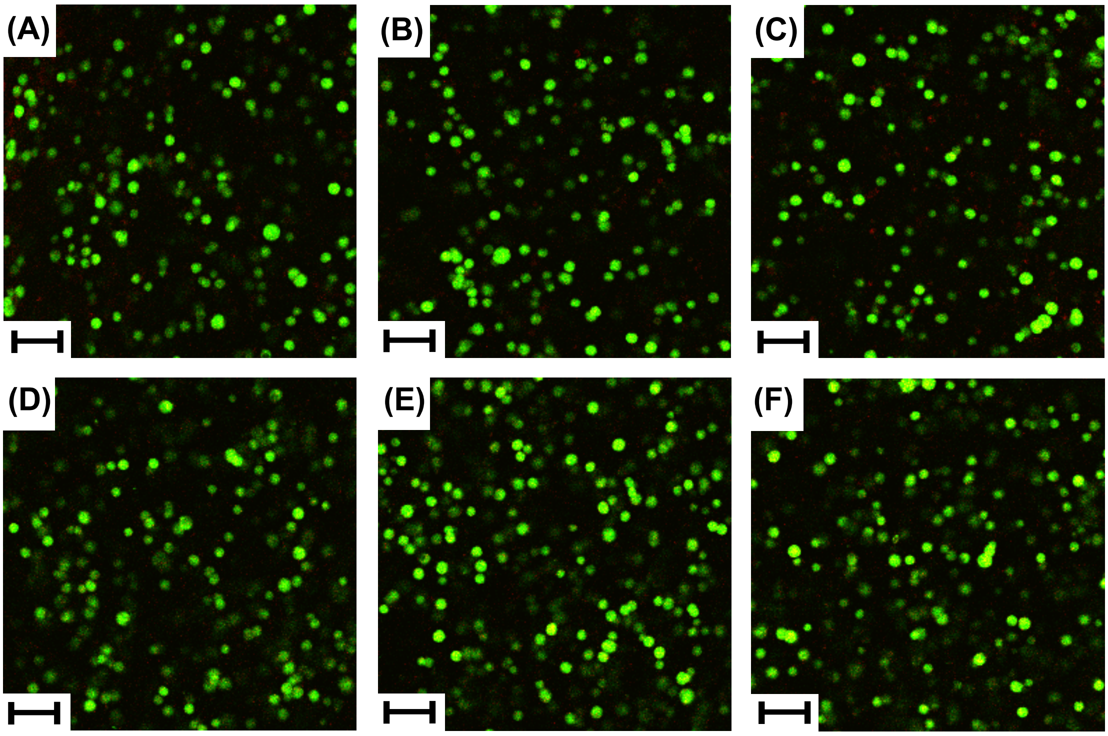

Fig. S8. hMSC viability in Scl2 hydrogels. LIVE/DEAD® Viability/Cytotoxicity assay on hMSCs cultured for 6 weeks in hydrogels. Representative confocal images of cells in (A) MMP7-HHACS-Scl2, (B) MMP7-HHACS-lowRGDS-Scl2, (C) MMP7-HHACS-highRGDS-Scl2, (D) ScrMMP7-HHACS-Scl2, (E) ScrMMP7-HHACS-lowRGDS-Scl2, and (F) ScrMMP7-HHACS-highRGDS-Scl2 hydrogels (scale bars are 50 μm).

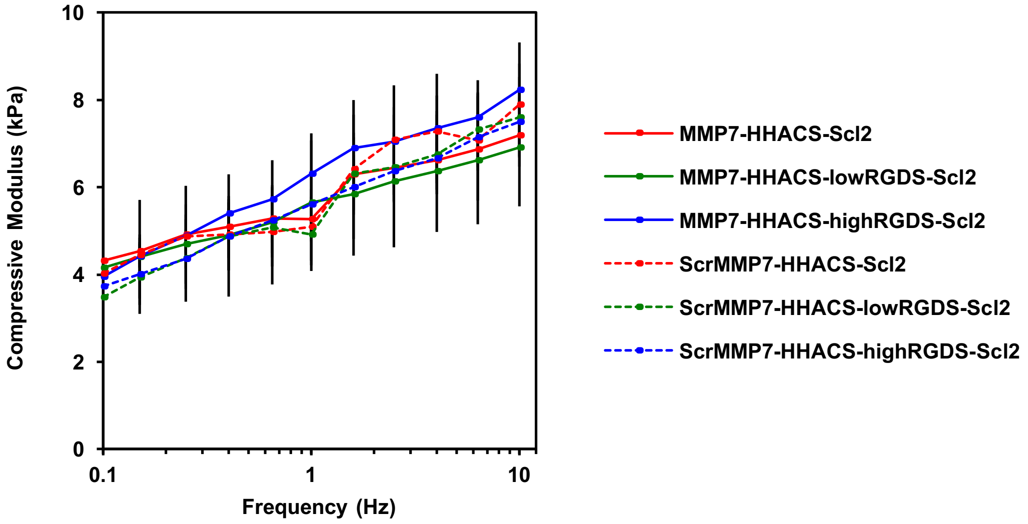

Fig. S9. Dynamic mechanical analysis (DMA). Elastic compression moduli of cell–seeded Scl2 hydrogels compressed to 10% strain at 0.5% strain/min 0.1 to 10 Hz after 6 weeks of culture. Values represent means ± SD (n = 3 for each donor; 3 different bone marrow–derived hMSC donors).

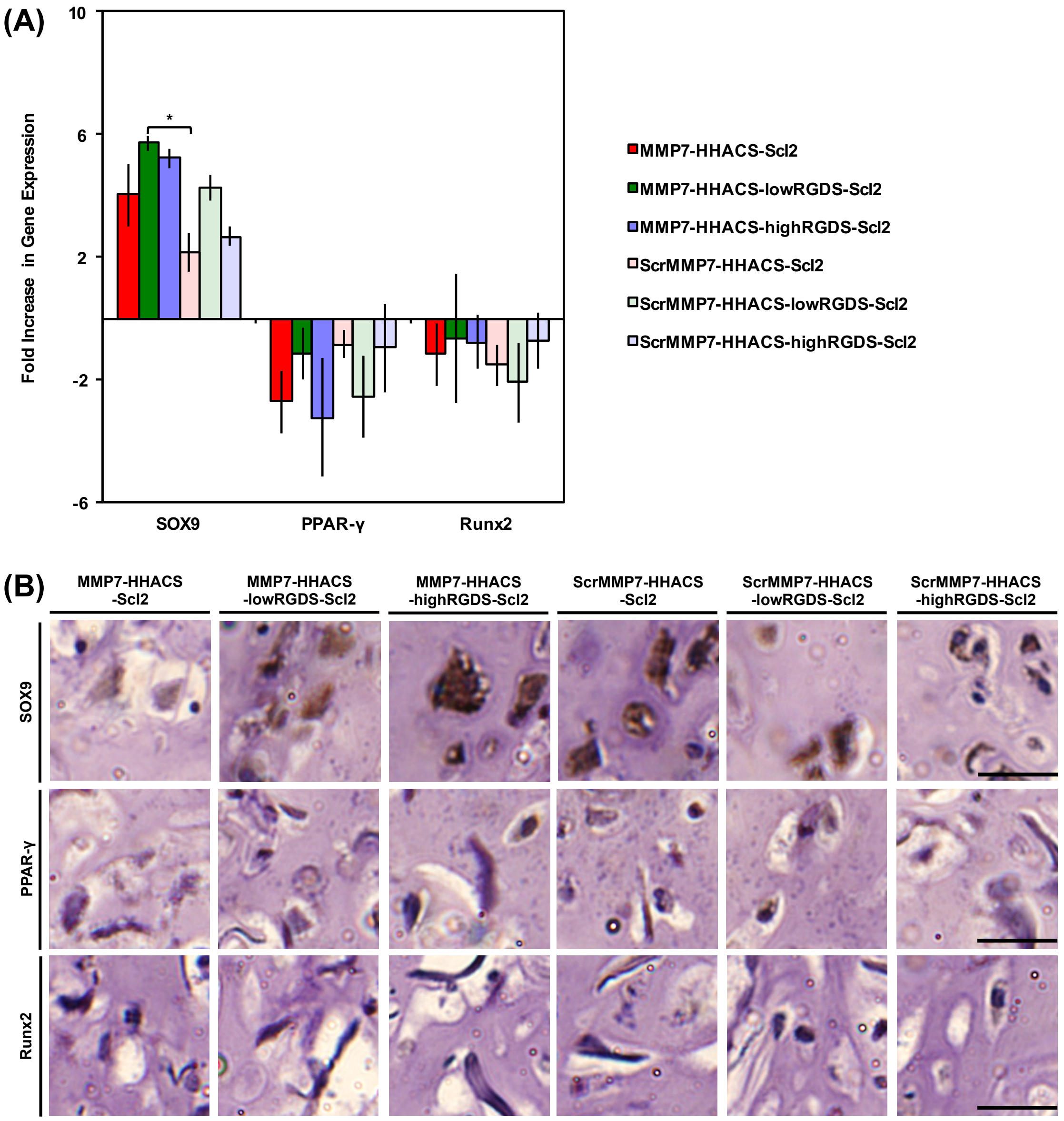

Fig. S10. (A) hMSC gene expression in Scl2 hydrogels. SOX9, Runx2, and PPAR-γ gene expression of hMSCs encapsulated in hydrogels after 2 weeks in culture, as analyzed using the ΔΔCt method. Data presented as a fold difference relative to undifferentiated hMSCs (calibrator) prior to encapsulation and normalized to GAPDH (housekeeping gene). Values represent means ± SD. $*p < 0.05$ (n = 3 for each donor; 3 different bone marrow–derived hMSC donors). (B) High magnification images showing representative immunohistochemical examination of hMSC–seeded hydrogels after 14 days in culture. Hydrogels are stained for SOX9, PPAR-γ, and Runx2, respectively, from top to bottom. Scale bars are 100 μm.
